# Recent Developments in Free Energy Calculations for Drug Discovery

**DOI:** 10.3389/fmolb.2021.712085

**Published:** 2021-08-11

**Authors:** Edward King, Erick Aitchison, Han Li, Ray Luo

**Affiliations:** ^1^ Department of Molecular Biology and Biochemistry, University of California, Irvine, CA, United States; ^2^ Department of Chemical and Biomolecular Engineering, University of California, Irvine, CA, United States; ^3^ Department of Materials Science and Engineering, University of California, Irvine, CA, United States; ^4^ Department of Biomedical Engineering, University of California, Irvine, CA, United States

**Keywords:** binding affinity, free energy simulation, drug discovery, molecular dynamics, MM-PBSA, lie, alchemical simulation

## Abstract

The grand challenge in structure-based drug design is achieving accurate prediction of binding free energies. Molecular dynamics (MD) simulations enable modeling of conformational changes critical to the binding process, leading to calculation of thermodynamic quantities involved in estimation of binding affinities. With recent advancements in computing capability and predictive accuracy, MD based virtual screening has progressed from the domain of theoretical attempts to real application in drug development. Approaches including the Molecular Mechanics Poisson Boltzmann Surface Area (MM-PBSA), Linear Interaction Energy (LIE), and alchemical methods have been broadly applied to model molecular recognition for drug discovery and lead optimization. Here we review the varied methodology of these approaches, developments enhancing simulation efficiency and reliability, remaining challenges hindering predictive performance, and applications to problems in the fields of medicine and biochemistry.

## Introduction

Modern drug development requires screening over vast regions of chemical space to identify potential binders against a protein target. This approach is costly in time and material resources (DiMasi et al., 2016). Even after identification of potential ligands from initial screening assays, further refinement must be carried out to improve binding properties, ensure that off target effects are minimized, and optimize pharmacokinetic properties. Evaluation of binding free energies through virtual screening has shown promise in efficiently narrowing the chemical search space for candidate compounds and streamlining the process of lead compound optimization. Outside of the pharmaceutical field, binding affinity predictions find additional uses in protein engineering, and guide the rational design of mutations altering enzyme substrate/product specificity (Kaushik et al., 2018; Li Y. et al., 2019; Bhati et al., 2019; Ono et al., 2020; Chen et al., 2021), structural stability (Aldeghi et al., 2018; Jandova et al., 2018; Pourjafar-Dehkordi et al., 2019; Martin et al., 2020), and catalytic efficiency (Xue et al., 2019; Wang K. et al., 2020).

**FIGURE 1:**
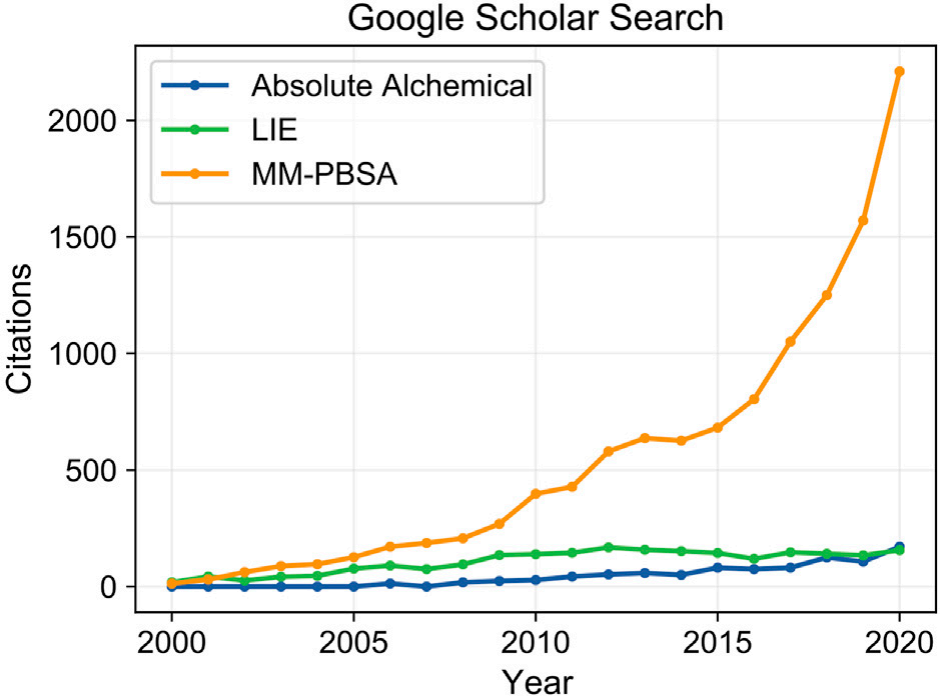
Citation counts for each method over the past 20 years. The development and utilization of molecular simulation to guide drug discovery has grown dramatically in recent years. The MM-PBSA method, which balances simulation rigor, high speed, and minimal setup complexity to allow high throughput screening, has seen extensive application reaching over 2,000 citations in 2020. Steep computational costs and challenges in generalizing protocols to work on broad sets of protein-ligand systems have limited the usage of absolute alchemical and LIE based approaches.

Here we discuss recent developments and applications of molecular dynamics to calculate absolute binding free energies in protein-ligand binding interactions. Through utilization of the Molecular Mechanics Poisson Boltzmann Surface Area (MM-PBSA) (Cheatham et al., 1998; Srinivasan et al., 1998; Kollman et al., 2000; Gohlke and Case, 2004; Yang et al., 2011; Miller et al., 2012; Wang et al., 2016; Wang et al., 2018a), Linear Interaction Energy (LIE) (Aqvist et al., 1994; Aqvist and Marelius, 2001; Aqvist et al., 2002; Gutierrez-de-Teran and Aqvist, 2012), and absolute alchemical methods (Kirkwood, 1935; Zwanzig, 1954; Kirkwood, 1967; Bennett, 1976; Straatsma and McCammon, 1991; Gilson et al., 1997; Boresch et al., 2003; Shirts, 2012), researchers are able to evaluate biomolecular interactions that drive molecular recognition at atomic resolution and derive accurate predictions for binding free energies. These methods rigorously account for conformational dynamics and solvent interactions that are key to protein-ligand interactions and absent in coarser-grained approaches such as ligand docking. The value in these methods for advancing drug discovery is highlighted by their widespread application. Within the last 20 years the number of citations for each method has grown from a small handful to several thousand, notably the MM-PBSA method was found in over 2,000 citations in the last year (Figure 1). These three methods differ in their treatment of solvent and required simulation data, either involving only the end point states of bound and unbound species, or demanding simulation of a complete binding pathway traversing intermediate states between the end points for determination of binding free energy. These differences result in trade-offs between predictive accuracy and computational cost that must be weighed by the user to select the best approach for their application. In this review, discussion of approaches for the calculation of relative binding free energies is skimmed over as having been recently reviewed elsewhere (Cournia et al., 2017; Song and Merz, 2020). We focus on describing the fundamental principles of each method, recent developments enhancing their usability by improving accuracy and computational efficiency, and successful applications in drug discovery projects.

## Free Energy Calculation Approaches

### Molecular Mechanics Poisson Boltzmann Surface Area

**FIGURE 2:**
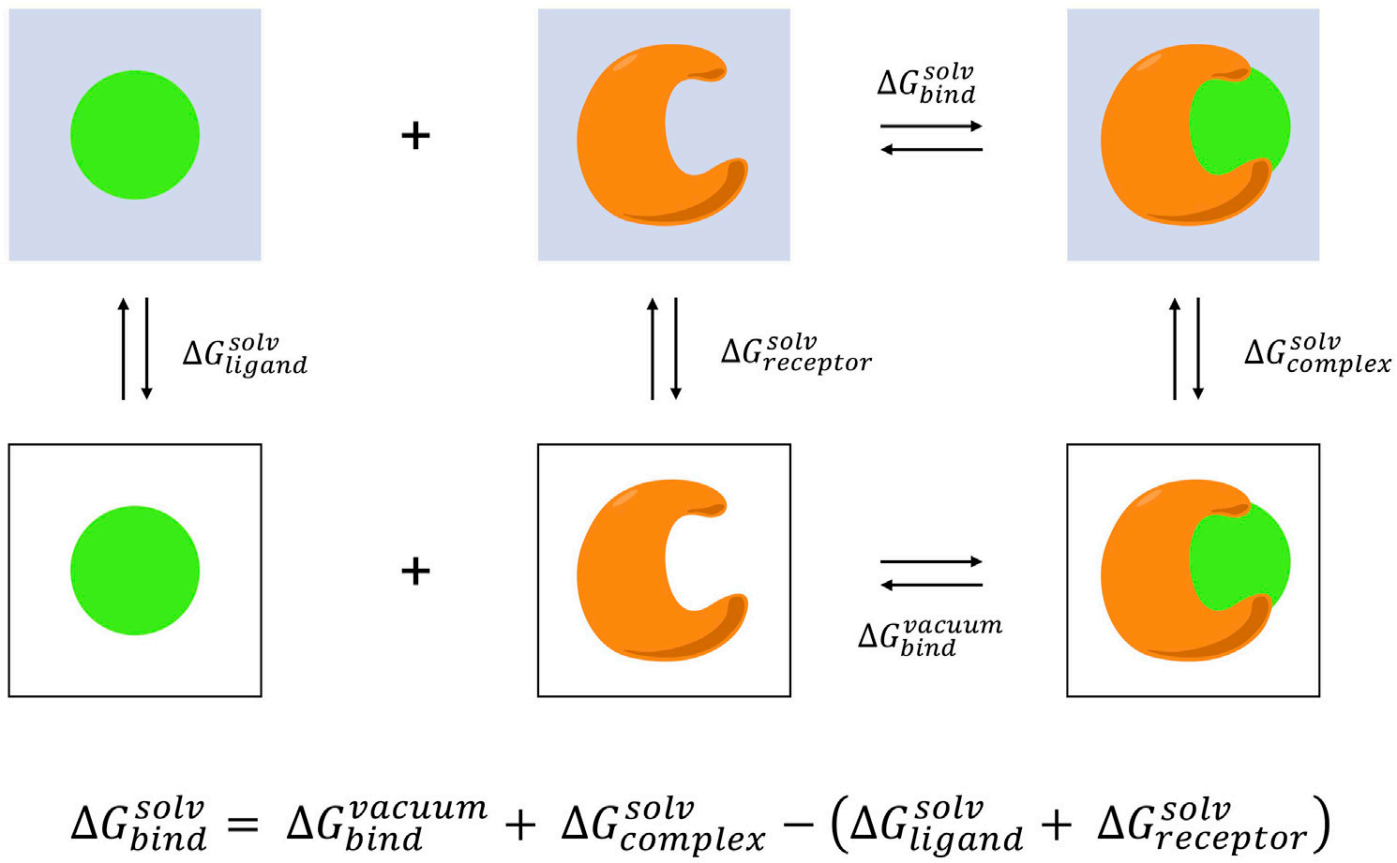
MM-PBSA thermodynamic cycle. The binding free energy in aqueous environment is calculated as the difference between the sum of binding in vacuum and solvating the complex with solvating the receptor and ligand individually. The information necessary to complete this cycle can be obtained by decomposing a single trajectory into the ensemble desolvated receptor, ligand, and complex configurations, and computing the solvation free energies for each state with the Poisson-Boltzmann equation. Normal mode analysis can be performed to determine the contribution of entropy to the binding process.

The MM-PBSA method as applied to small molecule binding is an end-point method estimating the binding free-energy difference between the protein-ligand complex and the separate unbound components, the complex, ligand, and protein alone (Sharp and Honig, 1990; Cheatham et al., 1998; Srinivasan et al., 1998; Kollman et al., 2000; Gohlke and Case, 2004; Yang et al., 2011; Miller et al., 2012; Genheden and Ryde, 2015; Wang et al., 2016; Wang et al., 2019a) (Figure 2). MM-PBSA provides a balanced approach characterized by improved rigor and accuracy over molecular docking, and with reduced computational demands compared to pathway methods such as alchemical transformations that require involved experimental setup to sample intermediate states through the decoupling of ligand interactions (Rastelli et al., 2010; Hou et al., 2011; Slynko et al., 2014; Sun et al., 2014). In addition to only requiring end-point data, a further approximation with MM-PBSA that enables efficient free-energy calculation is the utilization of implicit solvation. By coarse-graining solvent as a continuum with uniform dielectric constant the treatment of solvent interactions is greatly simplified. However, this may lead to difficulties modeling highly charged ligands and recent works have focused on minimizing these errors (Wang et al., 2019a).

Two main approaches are employed to generate the data for MM-PBSA binding energy predictions with both starting from molecular dynamics (MD) simulation in explicit solvent: multiple trajectories with the three components, complex, apo receptor, and ligand separately, or a single trajectory with the bound protein-ligand complex that is divided into the three components afterward (Kollman et al., 2000; Wang et al., 2016). MD is carried out with explicit solvation to maximize accuracy of conformational sampling, and frames are post-processed by removal of solvent and ion molecules. The converged trajectory is evaluated with each frame as an individual sample point to generate ensemble averages and uncertainty values for the energy quantities. The single-trajectory approach is favored for its straightforward implementation and cancellation of covalent energy errors as conformations for the complex and separated receptor and ligand are based on shared configurations. However, the single-trajectory method may not be optimal due to its reliance on the problematic assumption that ligand binding does not involve large-scale conformational changes (Lee and Olson, 2006; Wang et al., 2016). The multi-trajectory approach is better suited for binding events associated with large conformation changes, but is noted to produce noisier estimates and require longer simulation time to reach convergence as the complex and individual components can sample diverged conformations (Swanson et al., 2004; Yang et al., 2011).

The binding free energy between the ligand (L) and receptor (R) is defined as:

$$\Delta G_{bind}=G_{RL}-G_{R}-G_{L}$$

The difference in free energy between the complex and individual components can be decomposed into enthalpic (ΔH) and entropic (-TΔS) terms evaluating changes in bonding interactions and conformational disorder with binding. The enthalpic energy term can be approximated as the gas-phase molecular mechanics energy (ΔE_MM_) and solvation free energy (ΔG_solv_). The configurational entropy (−TΔS) can be estimated with the normal mode or quasi-harmonic analysis (Yang et al., 2011; Kassem et al., 2015), but is often omitted due to high computational cost and difficulty obtaining convergence.

$$\Delta G_{bind}=\Delta H-T\Delta S\approx \Delta E_{MM}+\Delta G_{solv}-T\Delta S$$

ΔE_MM_ is computed from the molecular mechanics force field and consists of the covalent energy (ΔE_covalent_), electrostatic energy (ΔE_elec_), and van der Waals dispersion and repulsion energy (ΔE_vdW_). The covalent term includes changes in bonds (ΔE_bond_), angles (ΔE_angle_), and torsion (ΔE_torsion_) energies.

$$\Delta E_{MM}=\Delta E_{\mathrm{cov}alent}+\Delta E_{elec}+\Delta E_{vdW}$$

$$\Delta E_{\mathrm{cov}alent}=\Delta E_{bond}+\Delta E_{angle}+\Delta E_{torsion}$$

ΔG_solv_ describes the contribution of polar and non-polar interactions to the transfer of the ligand from gas phase to solvent. The polar solvation component (ΔG_polar_) specifies the interaction energy of the solute’s charge distribution in the continuum solvent and is found by evaluation of the Poisson-Boltzmann equation (PBE) (Perutz, 1978; Warwicker and Watson, 1982; Bashford and Karplus, 1990; Davis and McCammon, 1990; Jeancharles et al., 1991; Gilson, 1995; Honig and Nicholls, 1995; Edinger et al., 1997; Luo et al., 1997; Luo et al., 2002; Sharp and Honig, 2002; Lu and Luo, 2003; Tan et al., 2006; Cai et al., 2009; Wang et al., 2009; Ye et al., 2009; Cai et al., 2010; Wang et al., 2010; Wang and Luo, 2010; Ye et al., 2010; Cai et al., 2011; Hsieh and Luo, 2011; Botello-Smith et al., 2012; Wang et al., 2012; Liu et al., 2013; Wang et al., 2013; Wang et al., 2017). The non-polar solvation term (ΔG_non-polar_) measures the energy from the solute forming a cavity in the solvent and the van der Waals interactions at the cavity interface between solute and solvent (Wagoner and Baker, 2006; Tan et al., 2007), so that the total solvation free energy can be expressed as:

$$\Delta G_{solv}=\Delta G_{polar}+\Delta G_{non-polar}$$

The basis of the PBE is the Poisson equation with dielectric distribution ε(**r**), electrostatic potential distribution φ(**r**), and fixed atomic charge density ρ(**r**), where each function is dependent on the solute atom position vector (**r**).

∇ε(r)∇φ(r)=−4πρ(r)

To account for electrostatic interactions from ionic salt molecules in the solution, the electrostatic potential (φ(**r**)) is solved with the PBE with the additional terms λ(**r**) representing the ion-exclusion function set to 0 inside the Stern layer and molecular interior and 1 outside, and salt-related term f(φ(**r**)) that depends on the electrostatic potential, the valence (z_i_), electron charge (e), bulk concentration (c_i_), and temperature (T), with summation over all ion types (i).

∇ ε(r)∇φ(r)+λ(r)f(φ(r))=−4πρ(r)

$$f\left(\varphi \left(r\right)\right)=4\pi \sum_{i}^{n}z_{i}ec_{i}\exp\left(-\frac{z_{i}e\varphi \left(r\right)}{k_{B}T}\right)$$

The PBE can be linearized for easier numerical computation under conditions where the ionic strength and electric field are both weak. The linear PBE equation includes the modified Debye-Hückel parameter (κ^2^), solvent dielectric constant (ε_solv_), and solution ionic strength (I) where I = z^2^c.

$$\nabla \;\varepsilon \left(r\right)\nabla \varphi \left(r\right)-\varepsilon_{solv}k^{2}\varphi \left(r\right)=-4\pi \rho \left(r\right)$$

$$k^{2}=\frac{8\pi e^{2}I}{\varepsilon_{solv}k_{B}T}$$

MM-PBSA is often used in tandem with the closely related Molecular Mechanics Generalized Born Surface Area (MM-GBSA) approach as both utilize the same set of inputs for the prediction of binding free energies with continuum solvation (Chen et al., 2016; Wang et al., 2019b). The difference between the methods lies in the calculation of ΔG_polar_ where the GB model is based on an analytical expression approximating the PBE. This leads to large speed improvements, but predictive performance is generally reduced compared to PBE, though this is system dependent (Genheden and Ryde, 2015; Chen et al., 2016). The GB equation is composed of terms describing solute atoms as spheres with partial charge (q), internal dielectric (ε) and solvent dielectric (ε_0_), distance between particles i and j (r_ij_), and the effective Born radius (α).

$$\Delta G_{GB}=-\left(\frac{1}{\varepsilon }-\frac{1}{\varepsilon_{0}}\right)\sum_{i,j}\frac{q_{i}q_{j}}{f_{GB}}$$

$$f_{GB}=\sqrt{r_{ij}^{2}+\alpha_{i}\alpha_{j}\exp\left(-\frac{r_{ij}^{2}}{4\alpha_{i}\alpha_{j}}\right)}$$

ΔG_non-polar_ has classically been determined as proportional to the solute’s solvent accessible surface area (SASA) (Wagoner and Baker, 2006; Tan et al., 2007) as:

$$\Delta G_{non-polar}^{SA}=\gamma *SASA+b$$

The surface tension constant (γ) describing the free energy of forming a cavity in water and the offset (b) are determined empirically and set as constants for all solute molecules. These variables are assigned as γ = 0.00542 kcal/mol-A^2^ and b = 0.92 kcal/mol in the AMBER package (Case et al., 2005; Miller et al., 2012; Case et al., 2020). Alternative methods with atom-specific surface tension constants have also been explored (Eisenberg and McLachlan, 1986; Ooi et al., 1987).

More updated methods to resolve ΔG_non-polar_ incorporate the van der Waals dispersion free-energy as a separate term, treating the process as two events where a cavity is created and the non-polar solute is inserted into the cavity (Tan et al., 2007). The separation of terms additionally allows individual scaling of the cavity formation and dispersion terms as a function of solute size. ΔG_cavity_ is calculated with similar linear regression as the classical ΔG_non-polar_ equation with SASA replaced with solvent accessible volume (SAV) and the attractive dispersion energy is computed through surface-integration. The updated scaling factors are set as γ = 0.0378 kcal/mol-A^3^ and b = -0.569 kcal/mol in the AMBER package (Case et al., 2005; Miller et al., 2012; Case et al., 2020).

$$\Delta G_{non-polar}^{CD}=\Delta G_{dispersion}+\Delta G_{cavity}$$

$$\Delta G_{cavity}=\gamma *SAV+b$$

### Molecular Mechanics Poisson Boltzmann Surface Area Developments and Benchmarks

Improvements to the MM-PBSA method include more rigorous treatment of the dielectric constants and electrostatic polarization for better predictive accuracy on highly charged ligands, faster PB solvers, extension to pKa calculation, and novel schemes for determination of entropy. Scaling of the solute dielectric constant to tune the screening of electrostatic interactions in the non-polar protein environment is found to have a critical, receptor-dependent role on predictive accuracy (Hu and Contini, 2019). Heterogenous dielectric values are applied to implicit membrane models where the dielectric is discretely varied with membrane depth (Greene et al., 2019), and with Gaussian dielectric to smoothly distribute the interface over protein cavities (Hazra et al., 2019). Integration of a Gaussian based model for molecular volume and surface area determination with the Gaussian dielectric distribution removes sharp surfaces separating the solute and solvent for a surface free approach to MM-PBSA calculation (Chakravorty et al., 2019). Electronic polarization effects can be incorporated through the use of polarizable force fields such as AMOEBA, this is implemented in the boundary integral PBE solver PyGBe (Cooper, 2019). Combination of the polarizable Drude oscillator force field with PBSA lowers RMSE from 2.5 kcal/mol with the standard CHARMM36 force field to 0.8 kcal/mol in calculation of solvation free energies for 70 molecules in addition to reducing errors in alanine scanning (Aleksandrov et al., 2018). Coupling PBE calculation with Monte Carlo sampling of protonation states is applied to estimation of protonation free energies leading to pKa values within 2.05 pKa units RMSE of experiment using the Drude-PB method and within ∼0.8 pKa units RMSE using PypKa. (Aleksandrov et al., 2020; Reis et al., 2020). There are also updates to the PBE solvers through geometric multigrid on CPU allowing massively parallel scaling to 100 CPUs and a grid size of 10^9^ (Womack et al., 2018) and GPU implementation leading to ∼100 times speed up compared to CPU (Qi and Luo, 2019). Introduction of analytical interface and surface regulation for the immersed interface method is proposed to improve stability and convergence and GPU implementation leads to 20 times speed up (Wei et al., 2019b). Regularization methods are investigated under the matched interface and boundary framework for proper treatment of charge singularities for higher numerical accuracy (Lee et al., 2021). Finally extensions of the harmonic average method are proposed for fully taking advantage of the dense data parallelism to enhance the performance of PBE solvers on GPU platforms (Wei et al., 2019a). Ensemble MM-PBSA calculation through use of multiple independent trajectories and maintenance of an explicit ligand hydration shell on the bromodomain-containing protein 4 system, a key regulator of transcription, showed robust reproducibility (Wright et al., 2019). Menzer et al. (2018) implement a confining potential on ligand external degrees of freedom and higher order cumulant expansion terms for average receptor-ligand interaction energies for more effective treatment of entropy.

A number of recent benchmarks identify best-practices to achieve optimal accuracy and directly compare MM-PBSA with other binding free energy prediction methods to highlight its advantages and disadvantages in drug discovery. When testing of MM-PBSA was performed on over 250,000 ligands for the GPCR superfamily following docking (Yau et al., 2019; Yau et al., 2020), utilization of a single energy minimized structure is found to be the most computationally efficient method for virtual screening. In prediction of binding free energies and correct binding pose from 55 protein-RNA complexes, MM-PBSA (r_p_ −0.510) shows slightly lower performance than MM-GBSA (r_p_ −0.557) (Chen F. et al., 2018). Molecular mechanics 3-dimensional reference interaction site model (MM-3D-RISM) is shown to have similar predictive performance as MM-PBSA, but differs in decomposition of polar and non-polar solvation energies (Pandey et al., 2018). Mishra and Koca (2018) investigate the effects of simulation length, VDW radii sets, and combination with QM Hamiltonian on MM-PBSA predictions of protein-carbohydrate complexes. The conditions with optimal agreement to experiment are found to be 10 ns simulation with the mbondi radii set, and PM6 DFT method with QM resulting in the highest correlation of 0.96. Entropic effects are further studied by Sun et al. (2018) through comparison of normal mode analysis (NMA) and interaction entropy on over 1,500 protein-ligand systems with varying force fields. The most accurate results are obtained with the truncated NMA method, but due to high computational costs the authors recommend the interaction entropy approach instead, and force field choice made only minor differences. Enhanced sampling methods including aMD and GaMD are compared to conventional MD with MM-PBSA on protein-protein recognition, although the enhanced sampling methods are beneficial in encouraging exploration of conformational space, they do not improve binding affinity predictions on the timescales tested (Wang et al., 2019b). The effect of including a small number of explicit water molecules and performing NMA for entropy calculation is examined for the bromodomain system (Aldeghi et al., 2017). Using a limited number of solvent molecules (∼20) and entropy estimate improved MM-PBSA accuracy, although performance does not surpass absolute alchemical approaches the results came at significantly lower compute requirements.

The ease of performing MM-PBSA analysis and balance of speed and accuracy make it a popular method to use as an initial filter to rank drug candidates. Estimation of binding affinities with MM-PBSA for small-molecule protein-protein interaction inhibitors is automated with the farPPI web server (Wang Z. et al., 2019) and prediction of changes in protein-DNA binding affinities upon mutation with the Single Amino acid Mutation binding free energy change of Protein-DNA Interaction (SAMPDI) web server (Peng et al., 2018). Furthermore, due to its reliability MM-PBSA is often used as a baseline comparison or in combination with alternative methods for higher performance. Machine learning methods based on extracting protein-ligand interaction descriptors as features from MD simulation are compared to MM-PBSA on the tankyrase system (Berishvili et al., 2019). Machine learning also accelerates pose prediction methods based on short MD simulation combined with MM-PBSA through the Best Arm Identification method to obtain the correct binding pose with minimal number of runs (Terayama et al., 2018). QM approaches allow more accurate consideration of nonbonded electrostatic interactions, but their usage is limited by high computational costs. This problem is addressed through fragment-based methods where localized regions of the protein-ligand system are treated with QM and the more global effects of solvation, entropy, and conformational sampling are evaluated through MM-PBSA analysis (Wang Y. et al., 2018; Okimoto et al., 2018; Okiyama et al., 2018; Okiyama et al., 2019).

### LIE

**FIGURE 3:**
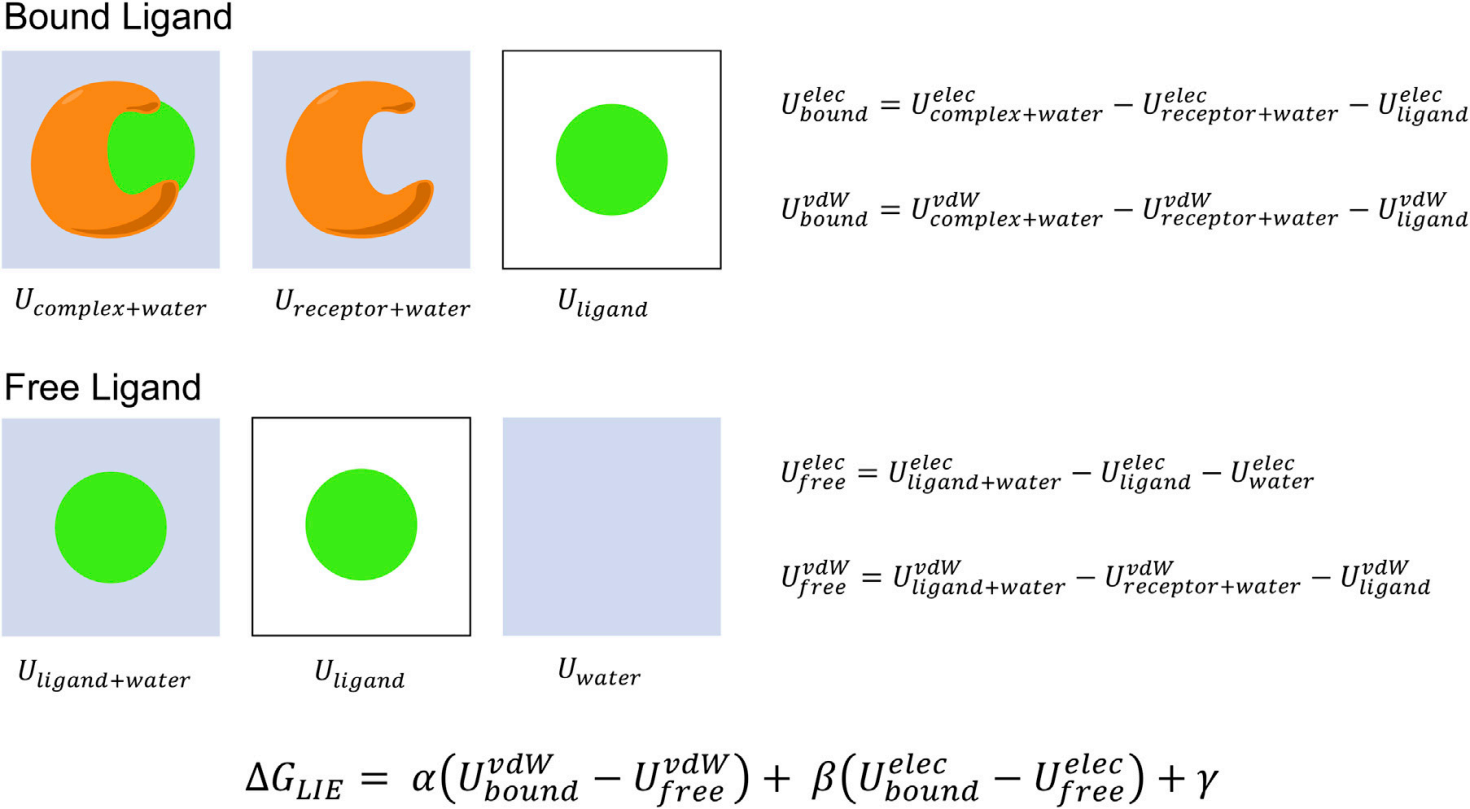
LIE binding free energy calculation. The binding free energy is computed from force field energy estimates of the differences in van der Waals and electrostatic energies for the ligand bound to the protein and free in solvent environment. The system dependent LIE parameters α and β are empirically determined and used to scale the non-polar and coulombic interaction energies to have minimal error with respect to available experimental data. The final term γ acts as an optional offset parameter to further tune the model. LIE requires no post-processing and can be completed from a single trajectory.

The Linear Interaction Energy (LIE) approach is another end-point method that predicts absolute binding free energies based on the change in free-energy from transferring the ligand from the solvated receptor-bound state to the aqueous free state (Aqvist et al., 2002; Gutierrez-de-Teran and Aqvist, 2012) (Figure 3).

$$\Delta G_{bind}\left(lig\right)=\Delta G_{solv}^{bound}\left(lig\right)-\Delta G_{solv}^{free}\left(lig\right)$$

This process considers binding in terms of the van der Waals (vdW) energy from creating the cavity in the target environment for the ligand and the electrostatic energy between the molecule and the environment. With that objective, LIE estimates ΔG_bind_ by an ensemble approach where two MD simulations are performed, with the ligand bound in the solvated protein and ligand free in solution, and the difference in VDW and electrostatic interactions between the ligand and environment in each case is measured (Aqvist et al., 1994; Hansson et al., 1998; Aqvist and Marelius, 2001).

$$\Delta G_{bind}=\left(\Delta G_{bound}^{polar}-\Delta G_{free}^{polar}\right)+\left(\Delta G_{bound}^{non-polar}-\Delta G_{free}^{non-polar}\right)=\Delta \Delta G_{bind}^{polar}+\Delta \Delta G_{bind}^{non-polar}$$

The molecular mechanics force field applied in MD provides potential energies (U) composed of polar and non-polar components that can be converted into free-energies. The linear response approximation where averages of the electrostatic interaction energies between the ligand and environment is utilized to determine the polar term. The second term ${\langle U_{lig-env}^{elec}\rangle }_{off}$ representing the potential electrostatic energy from conformations sampled with interactions between ligand and environment turned off is a negligible constant, and is generally ignored (Gutierrez-de-Teran and Aqvist, 2012).

$$\Delta G_{solv}^{elec}=\frac{1}{2}\left\{{\langle U_{lig-env}^{elec}\rangle }_{on}+{\langle U_{lig-env}^{elec}\rangle }_{off}\right\}=\frac{1}{2}{\langle U_{lig-env}^{elec}\rangle }_{on}$$

The scaling factor ½ is replaced with the variable β, and the polar component for LIE free-energy calculation considering bound and free ligand simulation is:

$$\Delta G_{bind}^{polar}=\beta \left({\langle U_{lig-env}^{elec}\rangle }_{bound}-{\langle U_{lig-env}^{elec}\rangle }_{free}\right)=\beta \Delta \langle U_{lig-env}^{elec}\rangle$$

Non-polar interactions including hydrophobic packing and van der Waals interactions are derived from the Lennard-Jones potential force field term. Due to the observed linear correlation of solvation free energies for non-polar compounds with solute size, and similar linear scaling for average van der Waals interaction energies with solute size, LIE assumes that average van der Waals energies can be directly employed to capture non-polar binding contributions with a similarly formed estimate as the polar component (Aqvist et al., 1994).

$$\Delta G_{bind}^{non-polar}=\alpha \left({\langle U_{lig-env}^{vdW}\rangle }_{bound}-{\langle U_{lig-env}^{vdW}\rangle }_{free}\right)=\alpha \Delta \langle U_{lig-env}^{vdW}\rangle +\gamma$$

The set of three empirical parameters: α to scale the vdW interaction energies (Wang et al., 1999), β to scale coulombic interaction energies (Åqvist and Hansson, 1996; Hansson et al., 1998), and γ as an optional offset constant (Almlof et al., 2004), are all freely tunable. These parameters are known to be system dependent and must be calibrated based on available experimental data (Almlof et al., 2007; van Dijk et al., 2017). Scaling of the model parameters is assumed to account for factors known to impact ΔG_bind_ but that are not explicitly declared including intramolecular energies, entropic confinement, desolvation effects, etc. The completed LIE estimation is based on force-field averaged energies and enables calculation of binding free energies solely through sampling of potential energies between the ligand and solvent or protein environments without post-processing

$$\Delta G_{bind}=\alpha \Delta \langle U_{lig-env}^{vdW}\rangle +\beta \Delta \langle U_{lig-env}^{elec}\rangle +\gamma$$

#### LIE Developments and Benchmarks

As the least computationally expensive method, LIE is uniquely suited for high-throughput screening and recent efforts are devoted toward the direction of improving predictive accuracy, even if the calibrated parameters are system dependent. To this end, multiple alterations to the base LIE protocol are proposed to more rigorously account for polar and entropic interactions by including additional terms, combining LIE results with PBSA (Huang et al., 2020) or alchemical calculations, and utilizing ensemble docking poses with iterative LIE models. The extended linear interaction energy method (ELIE) introduced by He et al. includes the PBSA terms for the polar solvation energy, non-polar solvation energy, and entropic contribution and individual scaling factors for each (He et al., 2019). Performance of ELIE in the Cathepsin S D3R 2017 Grand Challenge is found to show improved RMSE (1.17 kcal/mol) compared to MM-PBSA (3.19 kcal/mol) (He et al., 2019). Further benchmarking on 8 drug targets with a series of congeneric ligands to examine the application of ELIE to drug lead optimization demonstrates that ELIE (0.94 kcal/mol RMSE) can approach the accuracy of Free Energy Perturbation (FEP)/Thermodynamic Integration (TI) (1.08/0.96 kcal/mol RMSE) methods when using receptor-specific parameters. The authors find that 25 ns MD simulations show optimal accuracy as it generally decreases with longer simulation (Hao et al., 2020). The performance of LIE in host-guest systems is also evaluated on 4 host families (cucurbiturils, octa acids, β-cyclodextrin) with an array of 49 chemically diverse guests. The base LIE is modified to include host strain energy, and parameters are found to be transferable between the different host systems, notably resulting in binding predictions with RMSE below 1.5 kcal/mol through only a few nanoseconds of simulation (Montalvo-Acosta et al., 2018). Ngo et al. estimate HIV-1 protease inhibitor binding affinities with a modified LIE that includes a polar interaction term obtained from PBE, training on 22 samples and testing on a set of 11 ligands demonstrates good performance with 1.25 kcal/mol RMSE and 0.83 Pearson correlation (Ngo et al., 2020a). Proteins with flexible active sites may bind ligands in multiple orientations, this requires estimation of binding affinity from multiple poses weighted by their frequency to account for the contributions from each potential binding mode. Rifai et al. evaluate binding of inhibitors to malleable Cytochrome P450s with an iterative weighing approach where each training compound is sampled with multiple simulations starting from different binding poses and LIE parameters are determined from Boltzmann weighing individual trajectory results (Rifai et al., 2020). Further accuracy is obtained by combining LIE with alchemical simulations to consider the ligand solvation free energies. Direct comparison of LIE with MM-PBSA on the SIRT1 system with a set of 27 inhibitors finds that both methods produce comparable Pearson correlations of 0.72 for LIE and 0.64 for MM-PBSA indicating good predictive value in ranking inhibitors, LIE is advantageous in requiring shorter simulation due to slow convergence of the MM-PBSA polar term (Rifai et al., 2019). The two-domain LIE (2D-LIE) approach is introduced to predict the binding free energy between protein domains and applied to computing cellulase kinetics (Schaller et al., 2021).

### Absolute Alchemical Simulations

**FIGURE 4:**
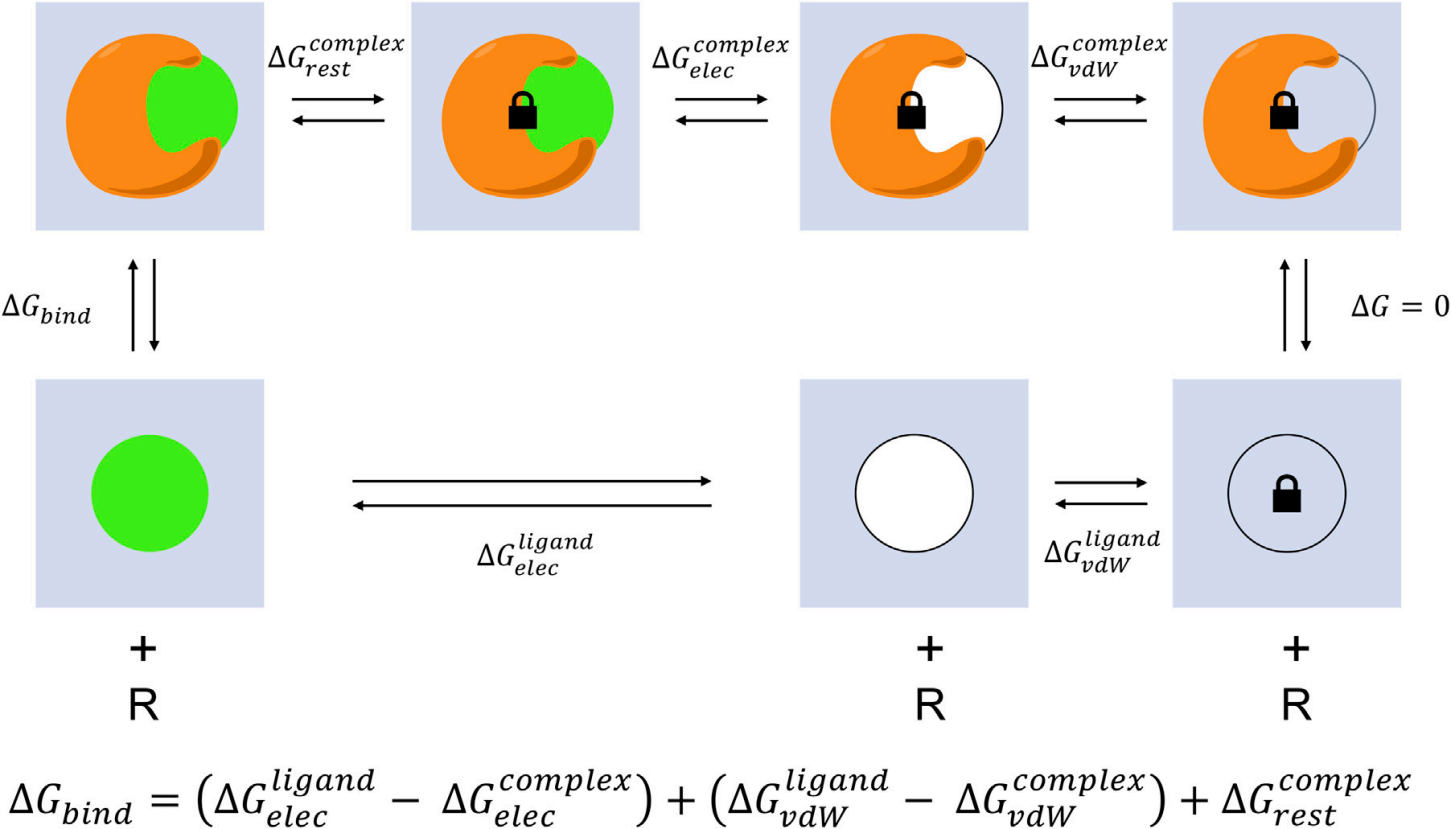
Absolute alchemical simulation thermodynamic cycle. Two trajectories are completed to model the unbinding process. The simulations start from the complex of protein-ligand bound and end with receptor and unbound ligand (top track), and from ligand alone in solvent to ligand removed (bottom track). The ligand is transformed through a series of unphysical states to decouple electrostatic and van der Waals interactions with the surrounding environment to reach the final state where it no longer interacts with the initial system. The binding free energy prediction is the sum of the coulombic and non-polar energies involved in the transformation eliminating protein-ligand interactions. A restraint is typically included to prevent the ligand from exiting the active site while the binding interactions keeping the protein and ligand together are scaled off in order to aid convergence, this is corrected for with an additional transformation progressively turning on the restraints for the complex track and an analytical correction for the ligand track.

End-point free energy prediction methods generally lack the ability to account for entropic and solvent effects, which play significant roles in protein-ligand interactions (Mobley and Dill, 2009), except for methods that explicitly compute end-state free energies such as the Mining Minima method (Head et al., 1997; Luo et al., 1999; Luo and Gilson, 2000; Mardis et al., 2001; Chen et al., 2004; Chang et al., 2007; Moghaddam et al., 2011). Capturing receptor conformation changes driven by ligand binding, water-mediated hydrogen-bonding, or solvent exchange that occurs as the ligand crowds the binding pocket are critical to rigorously estimate the free energy difference between the ligand bound and unbound states (Mobley et al., 2007). Pathway simulations tracking the MD trajectory of the ligand binding or unbinding event enable the computing of these effects, but come at high computational cost and increased simulation complexity (Woo and Roux, 2005; Lee and Olson, 2006; Gan and Roux, 2009) (Figure 4). The most direct approach to account for entropy and solvent effects in binding would be to simulate the receptor (R) and ligand (L) together and count the frequency of bound (RL) and unbound (R + L) conformations.

R+L⇌RL

The ratio of bound to unbound states is an equilibrium constant (K_eq_) that can be input into the Gibbs free energy equation where the Boltzmann constant (k_b_) and temperature (T) are multiplied with the natural log of K_eq_ to calculate the binding free energy (ΔG_bind_).

$$K_{eq}=\frac{\left[RL\right]}{\left[R\right]\left[L\right]}$$

$$\Delta G_{bind}=-k_{b}T\ln K_{eq}$$

In practice, it is not possible to estimate the equilibrium constant as the binding and unbinding events rarely occur within the timescales accessible with current simulation methods, leading to insufficient sampling. To bypass this sampling limitation, alchemical approaches modeling the gradual decoupling of electrostatic and van der Waals interactions between the ligand and receptor have been utilized to simulate the transition between ligand bound and unbound states without the need to physically capture the process (Zwanzig, 1954). The basis of this calculation is the thermodynamic cycle describing in one leg the removal of ligand from the complex, and in a parallel leg the removal of the ligand from solvent (Boresch et al., 2003). The end states with receptor alone and solvent alone interconvert with zero free energy difference as the ligand is absent from both systems, leaving the last transition between ligand in solvent to ligand bound to receptor solvable with knowledge of the free energy costs in transferring the ligand out of the receptor and out of solvent. This is typically performed through the Zwanzig equation also known as Exponential Averaging (EXP) or Free Energy Perturbation (FEP).

$$\Delta {\text{G}}_{\text{AB}}=-k_{b}T\ln{\langle -\frac{1}{k_{b}T}\left(U_{B}-U_{A}\right)\rangle }_{A}$$

EXP calculates the difference in potential of the end states using the ensemble of one simulated end state; however, this method is susceptible to bias in the free energies estimated due to poor phase space overlap of the end states (Lu et al., 2003).

Since free energy is a state function, its difference between states in the closed thermodynamic cycle is independent of the pathway taken, this includes non-physical intermediates that cannot be observed experimentally. The sampling of non-physical intermediate states is described by the parameter λ spanning from 0 where no perturbation has occurred to 1 where the ligand is fully decoupled from the environment and gives rise to the name alchemical. A drawback of the approach is the need for many intermediate states to guarantee accuracy of the simulation. The potential energies are computed for each intermediate state, and the free energy differences are calculated through thermodynamic integration by evaluating the integral of the ensemble averaged derivatives of potential energy with respect to λ (Kirkwood, 1935; Kirkwood, 1967; Shirts and Pande, 2005; Bruckner and Boresch, 2011b; a;de Ruiter et al., 2013).

$$U\left(\lambda \right)=\lambda U_{0}+\left(1-\lambda \right)U_{1}$$

$$\Delta G=\int_{0}^{1}<\frac{dU_{\lambda }}{d\lambda }{>}_{\lambda }d\lambda$$

Standard alchemical transformations are carried out in two stages, first with scaling ligand atom partial charges to model decoupling of electrostatics, and next with the van der Waals interactions (Shirts, 2012; Klimovich et al., 2015). These two transformations are performed separately to avoid singularity artifacts that arise from atomic overlap created by strong attractive electrostatic interactions drawing atoms lacking steric bulk over others (Beutler et al., 1994; Klimovich et al., 2015). It is also necessary to utilize an alternative “softcore” Lennard-Jones potential coupled to the λ window during the van der Waals scaling. Linear scaling with the standard Lennard-Jones potential leads to numerical instabilities at λ endpoints due to the severe repulsive forces calculated on overlapping atoms and contributes to poor phase space overlap with neighboring windows (Steinbrecher et al., 2007; Steinbrecher et al., 2011). An example “softcore” potential is illustrated as a function of the λ window and configuration (x), and contains the tunable parameters α, m, n, and standard terms for the distance where the pair-wise potential is 0 (σ) and the distance separating the atoms (r) (Hornak and Simmerling, 2004; Steinbrecher et al., 2011; Giese and York, 2018).

$$U\left(\lambda ,x\right)=4\varepsilon \lambda^{n}\left[{\left(\alpha {\left(1-\lambda \right)}^{m}+{\left(\frac{r}{\sigma }\right)}^{6}\right)}^{-2}-{\left(\alpha {\left(1-\lambda \right)}^{m}+{\left(\frac{r}{\sigma }\right)}^{6}\right)}^{-1}\right]$$

Further considerations involving the direction of the alchemical transformation, the utilization of restraints, the treatment of charge neutralization, λ window scheduling, procedure to select data samples that are both uncorrelated and equilibrated, and method to calculate free energy differences between the intermediate states must be made to ensure simulation stability and minimize variance in final free energy determination with the alchemical perturbation. The above factors all play some roles in the accuracy of the simulated free energies but are often not easy to decide *a priori*. Sampling of the physiologically relevant binding pose is essential to obtaining accurate values, initializing the alchemical transformation from an experimentally determined complex and modeling ligand decoupling generally maintains the ligand in the most applicable configurations (Klimovich et al., 2015). Theoretically there should be no difference beginning from the opposite end point state with an empty active site and having the ligand grown in; however, this may require longer simulation time as the ligand can easily get trapped in local minima away from the true binding pose and sample irrelevant states. The ligand may leave the binding pocket as the interactions with the receptor are scaled, hindering convergence (Mobley et al., 2006).

This is prevented by attaching restraints, which are later corrected for with an additional penalty term, to hold the ligand in the binding pocket. Two types of restraint schemes are common, the first involves imposing a single virtual bond between the ligand and receptor which is analytically corrected for by the formula

$$\Delta G_{restra\mathrm{int}}=-k_{b}T\ln\left[\frac{8\pi^{2}V^{0}K_{r}^{1\text{/}2}}{{\left(2\pi k_{b}T\right)}^{1\text{/}2}}\right]$$

where V^0^ is the standard state volume and K_r_ the force constant (Roux et al., 1996; Gilson et al., 1997; Mann and Hermans, 2000; Harger et al., 2017). An alternative restraining approach, the 6DOF method introduced by Boresch et al. (Boresch et al., 2003), enforces stricter adherence to a defined pose through one distance, two angular, and three dihedral restraints. Restraining the ligand to a single orientation expedites convergence, but may frustrate sampling of appropriate conformations not directly captured in the crystal structure, leading to overestimation of binding affinities (King et al., 2021). The 6DOF restraint correction is calculated with the following equation

$$\Delta G_{restra\mathrm{int}}^{6DOF}=-k_{b}T\ln\left[\frac{8\pi^{2}V^{0}{\left(K_{r}K_{\theta A}K_{\theta B}K_{\phi A}K_{\phi B}K_{\phi C}\right)}^{\frac{1}{2}}}{r_{a,A,0}^{2}\sin\theta_{A,0}\sin\theta_{B,0}{\left(2\pi k_{b}T\right)}^{3}}\right]$$

where r_a,A,0_ is the restrained distance, θ_A,0_ and θ_B,0_ are the two restrained angles, and K’s are the force constants (Boresch et al., 2003).

The transformation of charged ligands demands corrections to maintain neutrality in the simulation box as the ligand partial charges are scaled (Lin et al., 2014). Due to the usage of periodic boundary conditions, excess charges are propagated through all cells and cause errors in charge distribution (Hunenberger and McCammon, 1999; Anwar and Heyes, 2005; Hub et al., 2014). This issue can be managed by performing the partial charge scaling simultaneously on a specified counter-ion (Dixit and Chipot, 2001; Wallace and Shen, 2012; Chen W. et al., 2018), or through the correction scheme introduced by Rocklin et al. (Rocklin et al., 2013) based on an additional PB calculation to account for periodic finite-size effects.

The number and length of λ windows governs the variability of the free energy calculation (Shirts and Pande, 2005; Pham and Shirts, 2011). Increased sampling reduces the variance, but may not be worthwhile due to the added simulation costs. Rather than equally spacing the λ windows, a better strategy would be to more densely sample regions where transitions are non-linear near the end points of the van der Waals scaling stage and reduce sampling in more linear regions such as the electrostatic scaling. Datapoints from the beginning of each λ window are not yet equilibrated and sequential datapoints are autocorrelated, contamination with these energy values will distort the final free energy prediction (Chodera, 2016). Straightforward solutions to these problems would be to discard all data from the first half of the λ window and to only process energy values with large intervals (King et al., 2021). More sophisticated methods that aim to conserve as many datapoints as possible include the usage of automated equilibration detection based on reverse cumulative averaging (Yang et al., 2004) and subsampling of energies based on the calculated statistical inefficiency (Chodera, 2016), this can be performed with the pymbar (Shirts and Chodera, 2008) package written by the Chodera group. Lastly, thermodynamic integration is known to produce results with high variability due to the numerical integration over highly non-linear functions. The Bennett Acceptance Ratio (Bennett, 1976) (BAR) approach minimizes variance in the calculation of free energy by accounting for energies in neighboring states (Lu et al., 2003). The BAR calculation self-consistently solves for the free energy (C) that satisfies the relations where i and j are consecutive states and U is the potential energy from a selected state.

$$\Delta G=\ln\frac{\Sigma_{j}f\left(U_{i}-U_{j}+C\right)}{\Sigma_{j}f\left(U_{j}-U_{i}+C\right)}+C$$

ΔG=C

$$f\left(x\right)=\frac{1}{1+e^{x}}$$

However, this method can face the same issues as EXP/FEP if there is no overlap between neighboring states. This has been extended to the Multistate Bennett Acceptance Ratio (Shirts and Chodera, 2008) (MBAR) method addressing the critical issues in BAR and produces the lowest variance of all free energy estimators by using energy differences from all λ windows (Paliwal and Shirts, 2011).

#### Absolute Alchemical Simulations Developments and Benchmarks

A major impediment to the usage of alchemical simulations is their complicated setup and data processing for ligand decharging and vdW removal stages. Updates to the popular molecular dynamics packages NAMD (Chen H. et al., 2020) and AMBER (Lee et al., 2020a; He et al., 2020) enable GPU accelerated calculation of the $\frac{dU_{\lambda }}{d\lambda }$ term necessary for thermodynamic integration or energy cross terms for sampling conformations at different lambda values for MBAR computation. To support high-throughput alchemical screening and improved reproducibility, a number of software packages automate the experimental setup in preparing the simulation files with appropriately decoupled ligand topologies and output the final binding free energy prediction after processing the trajectories. These include the VMD plugin BFEE (Fu et al., 2018), the python tool BAT.py (Heinzelmann and Gilson, 2021) for AMBER, the CHARMM-GUI Free Energy Calculator (Kim et al., 2020), the web platform Biomolecular Reaction and Interaction Dynamics Global Environment (Senapathi et al., 2020) (BRIDGE) for GROMACS, and Flare (Kuhn et al., 2020).

Improvements in simulation efficiency have allowed faster sampling of protein-ligand binding conformations and exploration of longer timescales to more comprehensively capture the significant perturbations that occur from ligand decoupling in absolute alchemical simulations. Giese et al. (Giese and York, 2018) utilize the simple but effective parameter interpolated thermodynamic integration (PI-TI) scheme where intermediate lambda states are defined by scaling the ligand molecular mechanic parameters, this allows taking full advantage of the standard GPU accelerated MD integrators and existing Hamiltonian replica exchange methods (HREMD) without the need to implement any alchemical specific code. Validation of this study examined pKa predictions on a double strand RNA system resulting in an error within 1.2 pKa units. Monte Carlo methods based on making unphysical, Boltzmann weighed rotamer and torsion moves lead to greater conformational sampling and crossing of energy barriers that would necessitate substantial simulation time in MD. Pure MC (Cabeza de Vaca et al., 2018; Qian et al., 2019) and the hybrid MC/MD method Binding modes of Ligands Using Enhanced Sampling (BLUES) involving random ligand rotations, relaxation with MD, and final acceptance or rejection through nonequilibrium Monte Carlo are demonstrated to have greater binding mode sampling efficiency than standard MD. Hamiltonian replicas parallelize sampling backbone torsions of T4 lysozyme (Jiang et al., 2018) and solvent exchange in the cytochrome P450 binding site (Jiang, 2019) to speed convergence within 1 ns in the latter study. In cases where no reliable experimental structure with ligand bound is available, the generalized replica exchange with solute tempering (gREST) + FEP (Oshima et al., 2020) approach where protein-ligand interactions are weakened through simulation at high temperature to force refinement of ligand binding orientation or Alchemical Grid Dock (Minh, 2020) method can be performed to obtain high quality binding poses. Alternative lambda schedules aimed at reducing the number of intermediate windows to simulate without sacrificing low variance are introduced by Konig et al. (Konig et al., 2020) with enveloping distribution sampling and addition of a restraint energy distribution function in the screening of SARS-CoV-2 protease inhibitors (Li et al., 2020). Entropic bottlenecks caused by order/disorder transitions that inhibit convergence can be avoided by biasing the simulation with the integrated logistic function away from the transition regions (Pal and Gallicchio, 2019). Metadynamics methods utilizing a history dependent bias potential to drive sampling of unexplored conformations are used for the theophylline-RNA complex to get within 0.02 kcal/mol of experiment (Tanida and Matsuura, 2020). The Gaussian algorithm enhanced FEP (GA-FEP) method is used to guide the design of Phosphodiesterase-10 inhibitors and overcomes poor sampling by fitting the observed energies to a multivariate Gaussian distribution to extrapolate better converged energy values for downstream BAR calculation (Li Z. et al., 2019). Dual resolution models where the active site portions of the protein are modeled with full atom representation and other regions as coarse grained showed significant speedup with only minor loss in accuracy compared to the all-atom model for the lysozyme system binding with di-N-acetylchitotriose (Fiorentini et al., 2020). Sakae et al. (Sakae et al., 2020) demonstrate a modified alchemical approach starting with unrestrained ligand for broader sampling of binding poses and bypass the need to exhaustively enumerate all potential binding modes. The DeepBAR method applies generative modeling to construct sample conformations of the cucurbit[7]uril host-guest system for the BAR analysis without the need for intermediate state sampling to achieve higher computational efficiency (Ding and Zhang, 2021).

Advances in finite size and charge treatment schemes have improved accuracy in computing decharging energies, and new formulations for the evaluation of “soft-core” atoms lead to greater numerical stability and reduced variability in vdW removal. The poor representation of electronic polarization in molecular simulation makes binding affinity prediction for charged and titratable molecules challenging. Standard MD simulation is unable to model dielectric screening effects that alter the strength of ligand partial charges as it transitions between the polar solvent environment to the non-polar protein active site (King et al., 2021). We demonstrate that scaling the dielectric constant with the MBAR/PBSA continuum solvent model provides a convenient method to reproduce the effects of charge polarization without requiring any modification to the MD integrator. RMSE for the predicted binding affinities of inhibitors for urokinase plasminogen activator is reduced from 3.2 kcal/mol with standard alchemical simulation to 0.89 kcal/mol with MBAR/PBSA (King et al., 2021). The AMOEBA polarizable force field that incorporates electronic polarization through induced dipoles, atomic dipoles, and quadrupole terms is applied to the lead optimization of the MELK inhibitor IN17 (Harger et al., 2019). In the SAMPL7 TrimerTrip host-guest blind challenge, utilization of the AMOEBA force field shows excellent results with 7/8 samples having errors within 2 kcal/mol (Laury et al., 2018; Shi et al., 2020; Amezcua et al., 2021). The commonly used approach to maintain charge neutrality through co-alchemical ions is shown not to fully eliminate charge artifacts in periodic simulation boxes due to localized differences in electrostatic potentials and solvent densities for the distant ion and bound ligand (Ohlknecht et al., 2020b). Continuum-electrostatics calculations (Ohlknecht et al., 2020a) and the “Warp-Drive” (Ekimoto et al., 2018) method of simultaneously perturbing the protein-ligand complex and a distant unbound ligand are proposed to more accurately correct for finite-size effects. Difficulty in modeling the extraction of charged ligands from deeply buried binding sites with potential of mean force (PMF) methods is addressed with the AlchemPMF protocol where steric obstructions along the physical pathway are alchemically removed, resulting in improved binding free energy estimates on HIV-1 integrase and telomeric DNA G-quadruplex (Cruz et al., 2020). Li et al. (Li and Nam, 2020) develop the Gaussian repulsive soft-core potential to produce a linear hybrid Hamiltonian with respect to lambda to allow improved simulation efficiency over the standard separation-shifted potential that generates non-linear Hamiltonians. Extension of smooth-step soft-core potentials that are composed of monotonically increasing polynomial functions that have the desirable end-point values enable one-step alchemical transformations by overcoming the issues of end-point catastrophe, particle collapse, and large gradient jumps (Lee et al., 2020b).

Benchmarks of alchemical simulations demonstrate their utility and high accuracies. The SAMPL6 and SAMPL7 challenges (Rizzi et al., 2020) feature several entries examining alchemical approaches for CB[8] and tetra-methylated octa-acids host-guest systems with comparison to umbrella sampling (Han et al., 2018; Nishikawa et al., 2018), TrimerTrip host-guest system with comparison of AM1-BCC and RESP charge schemes (Huai et al., 2020), and evaluation of GAFF and CGenFF force fields (Khalak et al., 2020). Novel applications of alchemical simulation include the estimation of binding affinity change upon protein mutation through the ensemble thermodynamic integration with enhanced sampling (TIES) approach on the fibroblast growth factor receptor 3 (FGFR3), notably simulations without enhanced sampling are unable to capture conformational changes driven by protein mutation in the binding site (Bhati et al., 2019). PMF methods based on utilizing restraints to physically pull the ligand out of the binding site are directly compared to absolute alchemical approaches on the HIV-1 integrase system by Deng et al. (Deng et al., 2018), the final results show similar performance with absolute errors in the range of 1.6–4.3 kcal/mol for alchemical and 1.5–3.4 kcal/mol for PMF. The authors add that the alchemical approach supports simpler setup as they do not need to geometrically define the pathway for the ligand to exit the binding site. Loeffler at al. (Loeffler et al., 2018) validate alchemical simulation results from different software packages in the calculation of hydration free energies and determine that the tested packages (AMBER, CHARMM, GROMACS, and SOMD) produce consistent free energies. The scale of alchemical simulations is growing dramatically by harnessing cloud computing (Zasada et al., 2020). The report of massive-scale simulation of 301 HIV-1 integrase inhibitors on the IBM World Community Grid (Xia et al., 2019) highlights how the availability of distributed computing is enabling high-throughput FEP screening.

## Applications to Drug Discovery

Usage of free energy calculations is propelling pharmaceutical research. Work performed on a broad range of disease topics including understanding the mechanism for drug actions, optimizing binding affinities against target molecules, and identification of potential inhibitors from libraries demonstrate the importance of these tools. We survey practical applications of modern free energy calculations with attention on works with exemplary accuracy or data contribution, and further detail usage of free energy calculations on a range of biomedical targets. Recent work coupling simulation prediction with experimental validation is of exceptional interest. These studies provide a direct benchmark on the utilization of free energy methods rather than post-hoc analysis that may not generalize well to real-world problems. Secondly, efforts to complete screening campaigns and validation of free energy predictions contribute valuable datasets that can guide the development of future methods such as machine learning models that are prominently dependent on access to ample and diverse data.

Achieving chemical accuracy of below 1 kcal/mol error is still not typical with free energy calculations. Studies utilizing the end state methods MM-PBSA or LIE generally show high errors, a consequence of coarse graining solvent, electrostatic, and entropic interactions. Alternative metrics of Pearson correlation, measuring the linear association between simulation and experimental binding free energies, or Spearman correlation, where the ranked relationship between predicted and experimentally measured values are analyzed, are better suited to compare the performance of end state methods as they capture the ability of the models to select a small number of candidate drug compounds with the highest potential. For example in evaluating ligand binding to the purinergic platelet receptor P2Y_12_R, MM-PBSA shows absolute RMSE values over 6 kcal/mol from experimental measurements, but is still able to capture the correct trend in ligand binding affinities with Pearson correlation of 0.79 (Greene et al., 2016). In another work MM-PBSA shows RMSE for the Thrombin system at 4.26 kcal/mol, but highly accurate Pearson correlation of 0.86 (Wang et al., 2016). Several studies utilizing alchemical methods progress toward the threshold of chemical accuracy, and lay the groundwork for best practices to follow in future works. Aldeghi et al. achieve 1.54 kcal/mol RMSE with absolute binding free energy calculation on the bromodomain-containing protein 4 system through usage of Hamiltonian-exchange dynamics on top of standard sampling protocols (Aldeghi et al., 2017). Low MUE of 0.83 kcal/mol is achieved by Kuhn et al. in the prediction of relative affinities by carrying out the alchemical transformation in both directions with independent simulations to eliminate the effects of hysteresis (Kuhn et al., 2020). In studies where relative binding affinities are converted to absolute binding free energies, calibration of model predictions can be performed through scaling the average of the predicted binding free energies to equal the average of the experimental binding free energies (Wang et al., 2015; de Oliveira et al., 2019).

### SARS-CoV-2

The emergence of the severe acute respiratory syndrome coronavirus 2 (SARS-CoV-2) has caused a global health crisis with over 2 million deaths worldwide, compelling rapid drug development for potential therapeutics. Several major protein targets have been identified for inhibition of SARS-CoV-2 function and surveyed through molecular simulation for predicted binding affinity with repurposed and novel drugs, these include the RNA dependent RNA polymerase (Procacci et al., 2020; Wakchaure et al., 2020) (RdRp) that replicates the RNA genome, the main protease (Macchiagodena et al., 2020b; Ngo et al., 2020b; Chowdhury et al., 2020; Gupta et al., 2020; Gupta and Zhou, 2020; Jukic et al., 2020; Li et al., 2020; Milenković et al., 2020; Tejera et al., 2020; Aghaee et al., 2021; Bhardwaj et al., 2021) (3CL M^pro^) that mediates replication and transcription, the spike protein (Patil et al., 2021) involved in initiating infection by penetrating the host cell, S-adenosyl-methionine dependent methyltransferase (Sk et al., 2020) (nsp16) that adds the 5′-cap to mRNA essential for stability, envelope protein (Dey et al., 2020) that is involved in virion assembly and budding, Papain-like protease that functions in viral replication and immune response evasion (Bosken et al., 2020), and the host serine protease TMPRSS2 (Singh et al., 2020) that primes the spike protein. Alanine scanning is combined with MM-PBSA to identify the hot-spot binding residues GLU166 and GLN189 on M^pro^ as critical sites for inhibitors to target (Aghaee et al., 2021). Since only partial structures of the spike protein bound to the receptor protein angiotensin converting enzyme 2 (ACE2) exist, homology modeling is performed to structurally evaluate interactions mediating the spike protein ACE2 complex. MM-PBSA alanine scanning at the interface is utilized to determine the set of residues vital to the tight binding interaction. 5 residues disordered in the crystal structure, VAL445, THR478, GLY485, PHE490, and SER494, are identified to be crucial for ACE2 specificity (Sakkiah et al., 2020). By analyzing the binding poses obtained during MD simulation with hydroxychloroquine, Procacci et al. propose an inhibitor with improved potency for M^pro^ by restructuring polar contacts on the ligand for greater hydrophobic packing surface area (Procacci et al., 2020). El Hassab et al. perform fragment based drug design and link together generated fragments binding to RdRp (El Hassab et al., 2020). Potential vaccine candidates derived from B-cell and T-cell epitopes from the spike protein have their binding stability assessed through MD simulation (Das and Chakraborty, 2020).

The push for the rapid development of potential therapeutics for SARS-CoV-2 leaves many of these studies as exploratory in nature, predicting free energies of binding or ranking potential ligands without corresponding experimental data. These studies can be used in the future to benchmark these free energy techniques when the experimental data becomes available. However, we do want to highlight studies that have experimental data to compare with. One particular study evaluates the repurposing of FDA-approved drug molecules as M^Pro^ protease inhibitors using a workflow that combines docking, 100 ns molecular dynamics using a conventional force field, 5 ns molecular dynamics using a neural network derived pseudo-quantum mechanical/molecular mechanical force field (ANI), and finally MM-PBSA to refine the field of 1,615 molecules down to 9 molecules. Ten molecules out of 62 that were obtained after initial docking have experimental data for inhibition activity ranging in active (3), moderately active (3), and inactive (4). Out of the final set of nine selected molecules, two molecules are in the active range, one is moderately active, and no inactive molecules were selected. The study is cognizant of potential missed active molecules during the docking step and the loss of three active/moderately molecules during subsequent steps, but does not further evaluate the details for the loss of those molecules (Gupta and Zhou, 2020). An additional study looks at potential inhibitors for 3CL protease using Hamiltonian replica exchange and non-equilibrium alchemical simulations. The binding free energy of 21 potential inhibitors is calculated with four molecules having experimental data to compare to, three of the molecules having error within 2 kcal/mol and one with 5 kcal/mol (Macchiagodena et al., 2020b).

### Cancer

Anti-cancer therapeutics are a significant target for molecular simulation. Studies have evaluated potential small molecule or peptide inhibitors and interrogated the binding interactions of known drug-protein interactions. Novel data representation is applied to the investigation of tankyrase inhibitors targeting the Wnt pathway using a workflow that combines docking and machine learning scoring to filter through a library of 1.7 million potential inhibitors down to 174 molecules, Downstream QSAR screening using ADMET and physico-chemical features further reduces the number to 17 molecules. A subset of selected molecules is chosen to simulate using molecular dynamics calculating binding free energy using MM-PBSA and FEP with MBAR, which show reasonable agreement to experimental assays for two tested molecules (Berishvili et al., 2020). Another study of note is the calculation of binding free energy for inhibitors of the p53-MDMX/MDM2 interactions. The use of FEP with BAR results in mean absolute error (MAE) of 0.816 kcal/mol and root mean squared error (RMSE) of 1.064 kcal/mol for a set of five inhibitors targeting p53-MDMX interaction; however, for MDM2 and a set of 14 molecules the resulting MAE is 3.08 kcal/mol. Furthermore, the simulation of apo MDM2 structure to improve sampling of conformational states is used to generate a free energy landscape and free energy correction that improves the MAE to 1.95 kcal/mol and RMSE to 2.83 kcal/mol (Singh and Li, 2020). Additional studies overview the dissociation mechanism of GDP from Cdc42 which modulates cell migration and polarity (Kang et al., 2019). The AMOEBA polarizable force field is used to predict covalent and non-covalent inhibitors of fructose-bisphosphate aldolase A (Qi et al., 2019). The following works are reported to identify promising drug candidates, such as assessing the delivery of the antitumor agent paclitaxel via cell penetrating peptides (Wei et al., 2021), methotrexate analogs against drug resistant human dihydrofolate reductase (Rana et al., 2020), inhibitors for Adenosine A_3_ receptor (Lagarias et al., 2019), and peptide inhibitors against epidermal growth factor receptor (Tavakoli and Ganjalikhany, 2019) identify promising drug candidates. Simulation of lactate dehydrogenase A found overexpressed in the tumor environment (Jafary et al., 2019), kinesin spindle protein inhibition with (+)-morelloflavone (Ogunwa et al., 2019), chemosensitizing caryophyllene sesquiterpenes with doxorubicin on P-glycoprotein (Di Sotto et al., 2020), and inhibitor binding to the transcription silencing G-quadruplex element of the oncogene c-MYC (Chen B. et al., 2020) demonstrate the broad range of cancer targets. Other works of interest find altered inhibitor binding interactions with mutant anaplastic lymphoma kinase involved in lung cancer (Xiao et al., 2019), activated Cdc42-associated kinase 1 inhibitors (Granadino-Roldan et al., 2019), small molecules binding to the MUSHASHI family of RNA binding proteins (Minuesa et al., 2019), and negative regulation of STXBP4 on the Hippo pathway (Vargas et al., 2020).

### Neurodegenerative Diseases

Many studies have been performed on neurodegenerative disorders, with major focus on understanding Alzheimer’s disease. Thai et al. demonstrate how MM-PBSA can rapidly facilitate drug development by first predicting strong multipotent binding of CID 9998128 with Aβ_42_ peptide, Aβ_42_ fibrils, and β-secretase, then performing *in vitro* assays confirming that the compound inhibits Aβ fibril formation (Thai et al., 2018). Other works include the development of positron emission tomography/single photon emission computed tomography probes targeting Aβ for imaging (Kawai et al., 2018), rational design of anti-Aβ antibodies (Greene et al., 2018), small molecule inhibitors targeting Aβ fibrils (Ngo et al., 2019; Gupta and Dasmahapatra, 2020), β-sheet breaker peptides to interfere with amyloid fibril assembly (Shuaib et al., 2019), and structural modeling of aspartyl protease γ-secretase in complex with Aβ peptides (Hitzenberger and Zacharias, 2019). Further study of neurodegenerative disease involves fragment docking of reversible covalent inhibitors on calpain, a calcium dependent cysteine protease, whose overexpression has been correlated with neurodegenerative disorders (Zhang H. et al., 2019). The study uses FEP/λ-REMD as well as Site-Identification by Ligand Saturation to enhance the FEP calculations for a set of ten α-ketoamides. This results in an overall Pearson correlation of 0.85 compared to experiment; however, the correlation changes when categorizing these into covalent (0.84) and non-covalent (0.41) states, suggesting that only the covalent state is useful for inhibition (Zhang H. et al., 2019). Additional studies explore the identification and design of inhibitors against Caspase 8 involved in neural cell death (Ahmad et al., 2019) and evaluate novel 1,4-diazepane containing ligands as σ-receptor ligands with antioxidant and neuroprotective properties (Zampieri et al., 2020).

### Antibacterials and Antivirals

Innovation in the field of antibacterial discovery is driven by greater computing power allowing screening of larger compound libraries and more accurate assessment of binding affinities. This is necessary to overcome the challenges in the rise of infectious bacterial strains resistant to long-established antibiotics. MM-PBSA and bioassays are combined to guide discovery of antimicrobial compounds derived from petroleum ether extracts of *Peperomia blanda* on methylthioadenosine phosphorylase, highlighting an effective pipeline for isolating novel compounds and identifying potential targets (Al-Madhagi et al., 2019). Recent studies examine the effects of mutations on ligand binding to guanine riboswitches found to be potential antibacterial targets (Chen et al., 2019), the discovery of ligands targeting the virulence factor thermolysin from *Bacillus thermoproteolyticus* (Lamazares et al., 2021), selective inhibition of bacterial RNA polymerase via the nucleoside analog pseudouridimycin (Rabbad et al., 2019), berberine as an inhibitor of the multidrug efflux system MexXY-OprM found in aminoglycoside resistant *Pseudomonas* (Laudadio et al., 2019), inhibition of *Pseudomonas aeruginosa* quorum signaling and biofilm formation by targeting anthranilate-CoA ligase (Shaker et al., 2020), and inspection of cruzain inhibitor binding modes to develop more potent treatments for Chagas disease caused by *Trypanosoma cruzi* (Martins et al., 2018). The lung disease tuberculosis, caused by the bacteria *Mycobacterium tuberculosis*, is targeted with indolizines to inhibit enoyl[acyl-carrier] protein reductase (Venugopala et al., 2019), virtually screened for inhibitors against the polyphosphate kinase Rv2984 that carries out the essential reaction producing inorganic polyphosphate (Shahbaaz et al., 2019), and simulated with induced fit docking of outer membrane protein A (OmpATb) to discover inhibitors blocking pore-forming activity.

Development of effective antiviral drugs is necessary to combat pandemic viruses and emerging pathogenic agents. Antiviral therapies typically inhibit the machinery specifically involved in viral replication, but this selectivity is difficult to achieve as viruses hijack the host cell proteins for replication and drugs inhibiting these processes will also damage the host cells. A second obstacle is that the high rate of viral replication leads to rapid development in drug resistance. Structural evaluation of residues critical to forming ligand binding interactions such as hydrogen bonds and hydrophobic packing has provided insight to guide the design of inhibitors against HIV, influenza, Ebola, Dengue, and HPV. HIV infection leads to destruction of CD4^+^ T cells and development of Acquired Immunodeficiency Syndrome. There are currently no available treatments to cure HIV, but therapeutics can control HIV progression. Binding affinity predictions are applied to elucidate the binding mode of inhibitors targeting HIV-1 protease and understand mutant protease resistance mechanisms (Li et al., 2018; Wang R.-G. et al., 2020; Wang and Zheng, 2020). In particular, the work of Li et al. looks at ten inhibitors for the HIV-1 protease and compares MM-PB/GBSA methods for calculation of free energy using conventional and polarizable force fields as well as the scaling of the interior dielectric constant. The optimization of the dielectric constants results in an RMSE of 1.43 kcal/mol in MM-PBSA with correlation coefficient of 0.87 and an RMSE of 6.62 kcal/mol in MM-GBSA with a correlation of 0.78 (Li et al., 2018). Further work has targeted HIV-1 reverse transcriptase through large scale virtual screening to yield 4 compounds for experimental validation (Zhang et al., 2016). Influenza viral infection causes respiratory illnesses commonly called the flu that can lead to death. Work on antivirals to treat influenza includes utilizing amantadine probes to block influenza M2 proton channels to prevent virus replication (Tzitzoglaki et al., 2020), analyzing the effects of the hemagglutinin mutations on binding affinity to human receptors (Zhou et al., 2018), screening inhibitors for the PB2 protein of influenza RNA polymerase to inhibit generation of RNA primers essential for replication (Pham et al., 2020), optimizing neuraminidase inhibitors as lead compounds (Yu et al., 2019), and characterizing potential influenza polymerase inhibitors (Pérez-Sánchez et al., 2021). Ebola causes hemorrhagic fever and molecular interactions between the monoclonal antibody ADI-15946 and the Ebola GPcl receptor is studied (Hou and Zhang, 2020). Dengue is a tropical illness transmitted by mosquitoes, it is targeted with thioguanine small molecule inhibitors for the NS2B/NS3 protease (Hariono et al., 2019) and antiviral peptides binding to the envelope protein domain III (Isa et al., 2019). Therapies treating human papillomavirus targeting the E6 oncoprotein complex are also evaluated (Ricci-Lopez et al., 2019).

### Integral Membrane Proteins

The application of molecular dynamics in the prediction of binding affinities has expanded to include more challenging systems than typical proteins in aqueous environment. Successful simulation of integral membrane proteins usually includes the interactions of the protein with the surrounding lipid bilayer to model the non-polar setting. A major difficulty with the addition of the lipid molecules is their slow relaxation timescales impairing convergence. Corey et al. validate the application of free energy calculations with protein-lipid interactions and address the sampling issue by utilizing the coarse-grained Martini v2 force field (Corey et al., 2019). G-protein coupled receptors (GPCRs) are an important drug target due to their roles in recognizing extracellular signals and converting them into intracellular responses. Profiling of inhibitors against subsets of GPCRs including cannabinoid receptors (Jung et al., 2018; Ji et al., 2020; Yang et al., 2020), biphenyl scaffolds targeting free fatty acid receptors (FFAR1/FFAR4) involved in diabetes (Zhang X. et al., 2019), and CXCR4 (Shen et al., 2019) yield potential candidates for future experiment and insight into the interactions forming the binding interface. A study of the complement component fragment 5a receptor (C5aR) system explores the binding of two protein ligand inhibitors (Sahoo et al., 2018). Particular interest in this system arises from the simultaneous binding at multiple binding sites for a single ligand. These binding interactions are explored using MM-PBSA to elucidate important residues at differing sites. The residues predicted to have central impact are consistent with experimental mutational analysis (Sahoo et al., 2018). Additionally, GPCR dimerization is also studied with the TGR5 system through PMF computations using umbrella sampling and MM-PBSA. Computational results corroborate experimental FRET experiments in revealing residue hot spots at the 1/8 interface for dimerization inhibitors to target (Waschenbach et al., 2020). The mechanism of opioid pain suppression is examined through simulation of fentanyl with the *Gluoeobacter violaceus* ligand-gated ion channel. The study indicates that the fentanyl-binding induced conformational changes inhibit conduction through the channel (Faulkner et al., 2019).

### Nucleic Acids

Nucleic acids carry genetic data and regulate cell processes. Study of binding affinity predictions with DNA or RNA generally requires use of different force fields than those used for protein systems, but otherwise involves the same logic and data processing. Deng (Deng, 2019) compares the double decoupling and PMF approaches in the consideration of small molecule inhibitors in complex with G-quadruplex DNA, and finds that both approaches have errors within 2 kcal/mol of the experimentally determined binding free energies. Further work with DNA includes investigation of alkaloid binding to human telomeric G-quadruplex (Deng et al., 2019), umbrella sampling of catabolite activator protein to identify DNA binding induced conformation changes (Prabhakant et al., 2020), binding of the antiviral netropsin in the DNA minor groove (Zhang et al., 2018), examination of Z-DNA stability with modified cytosine bases (Vongsutilers et al., 2020), and binding recognition and allosteric mechanism of tryptophan-responsive regulatory protein-DNA (Mariadasse et al., 2020). Simulation of RNA features the prediction of riboswitch binding affinities (Hu et al., 2020) and *in silico* screening of aptamers targeting hepatitis B surface antigen (Sabri et al., 2019).

### Peptides

Peptides are short chains of amino acids that can function as therapeutic drugs, biosensors, and catalysts. Peptide sequences are often optimized to achieve a desired conformation capable of binding and inhibiting target proteins similarly to small molecule inhibitors, with the advantage that peptides can bind larger surface areas for greater specificity than small molecules. Simulation and *in silico* design of peptides has been performed to generate cyclic lead compounds against the hormone resistin through the alchemical method to optimize selectivity (Chi and Vargas, 2020), construct peptide inhibitors derived from the LEDGF/P75 protein against HIV1 integrase receptor (Kilburg and Gallicchio, 2018), investigate differences in free energy profiles of cell penetrating peptides on DOPC model lipid bilayers to characterize penetration efficiency (Her Choong and Keat Yap, 2020), and identify determinants for peptide binding to the PSM9 PDZ domain found in synaptic junctions (Harish et al., 2019).

### Metal Ions

Metal ions are coordinated by metalloproteins to catalyze reactions that are challenging to achieve with conventional organic chemical methods. They are important in oxygen transfer, redox reactions, and free radical capture. Additional roles for metal ions involve their ability to stabilize highly charged interactions such as those with the DNA phosphate backbone. Accurate representation of metal ions in molecular simulation is limited by the complications of polarization effects that are not captured in conventional force fields, suboptimal treatment of metal ion ligation to amino acid residues through restraints, and lack of well-tested force field parameters in comparison to those available for organic molecules. Even with these difficulties, study of metal ion binding with molecular simulation is constantly advancing. Jing et al. (Jing et al., 2018) utilize a polarizable force field to demonstrate that selective binding of Ca^2+^ and Mg^2+^ arises from many-body polarization effects. Improved parameterization of Zn^2+^ ions coordinating to Asp/Glu (Macchiagodena et al., 2020a) and His/Cys (Macchiagodena et al., 2019) enables more reliable simulation of zinc binding proteins, binding free energies of Mg^2+^ coordination with nucleoside di- and tri-phosphates such as ADP and ATP are studied with polarizable force fields (Walker et al., 2020), and an optimized 12-6-4 potential incorporating charge-induced dipole interactions allows accurate binding free energy calculation of Co^2+^ and Ni^2+^ to the enzyme glyoxalase I (Song et al., 2020). The impact of zinc ions on O6-methylguanine DNA methyl transferase DNA binding activity (Gharouni et al., 2021) and effects of sodium or calcium ions on calprotectin dimerization (Gheibi et al., 2019) is investigated.

### Biomedical Studies

A host of other biomedical applications outside the major categories discussed above have also been published in recent years. Cataract formation occurs through human γD-Crystallin aggregation and application of MD shows that the steroid lanosterol binds to hydrophobic surface regions near the C-terminal region to protect against dimerization (Kang et al., 2018). Inhibitors are identified to target the JAMM deubiquitinylases Rpn11 and CSN5 that remove covalently attached ubiquitins from proteins to regulate homeostasis (Kumar et al., 2018). Free energy calculation is used to study adenosine deaminase abnormal function as reported in rheumatoid arthritis (Tian et al., 2018), the ricin catalytic subunit found in the chemical weapon ricin (Botelho et al., 2020), the tyrosine phosphatase PTP-CPS4B involved in *Streptococcus pneumonia* metabolic signaling (Zaman et al., 2021), and clusters formed by uric acid and melamine that contribute to renal dysfunction (Chattaraj and Paul, 2020). *Escherichia coli* pathogenesis has been discovered to be driven by colonization factor I fimbriae binding to the Lewis A glycan epitope found in the small intestine (Mottram et al., 2018). The recognition mechanism for highly charged inorganic phosphate by phosphate binding protein has been studied with the polarizable forcefield AMOEBA to resolve the protonation state of the bound phosphate (Qi et al., 2018). Diabetes is a metabolic disease characterized by the inability to regulate blood sugar levels. Therapeutics have been explored through liver fructose 1,6-bisphosphate inhibitors to control gluconeogenesis-mediated overproduction of glucose (Proenca et al., 2020) and dipeptidyl peptidase 4 inhibitors to block degradation of incretins that stimulate decrease of blood glucose (Rahman et al., 2020). Half-life extension of insulin determir by complex formation with human serum albumin has been examined as a platform for drug delivery (Ryberg et al., 2020). Further studies assess the role of conformational changes in AcrB transporter contributing to multidrug resistance (Matsunaga et al., 2018), transport of cholesterol by the Aster-A protein (Moesgaard et al., 2020), and reduction of chronic inflammation by treatment with the peptide KCF18 binding to TNF-α and interleukin-6 (Jiang et al., 2019). Investigation into other areas includes adaptive immune response through toll-like receptor activity when bound to diprovocim (Su et al., 2019) and stability when complexed with lipopolysaccharide or neoseptin3 (Tafazzol and Duan, 2019), cooperative binding of heat shock protein 70 with piperine (Zazeri et al., 2020), agonists and antagonist binding to androgen receptor triggering different conformational changes (Azhagiya Singam et al., 2019), and the binding pathway of benzamidine to mutant trypsin protease through enhanced ligand sampling (Shao and Zhu, 2019).

### Non-Pharmaceutical Applications

Research in the fields of biotechnology and plant biology is also aided by molecular simulations. Studies of interaction mechanisms between the germination stimulant karrikins binding to their receptor KAI2 (Zhang et al., 2020) and 4-hydroxyphenulpyruvate dioxygenase inhibitors functioning as herbicides is reported (Fu et al., 2019). Manufacture of commercially valuable polyketides, secondary metabolites synthesized by multi-domain polyketide synthase complexes, has been investigated via comparison of thioesterase binding affinity to cyclized products to understand product release mechanisms of the antifungals amphotericin and nystatin (Wang et al., 2021). Binding free energy analysis is also used to elucidate the molecular basis of ketoreductase chain length and regiospecificity (Serapian and van der Kamp, 2019; Zhao et al., 2020). Potential insecticides targeting the ecdysone receptor to disrupt growth are identified (Horoiwa et al., 2019).

## Conclusion

Prediction of binding free energies via molecular simulation is playing a key role in accelerating drug development efforts by reducing the time and experimental effort required to establish functional pharmaceuticals. The growing number of successful applications highlights the utility of these computational methods. MM-PB/GBSA is recommended for initial stages of virtual screening where ranking of large number of candidates is performed due to its balance of improved reliability over molecular docking and speed. LIE can be alternatively used when the number of candidates is extremely high and simulation speed is prioritized as it bypasses the post-processing steps required for MM-PB/GBSA at the cost of some accuracy. In later stages of drug optimization where fewer simulations are necessary due to a narrowed range of candidates, alchemical methods are suggested due to their greater rigor in considering interactions with explicit solvent and conformational entropy. Challenges in reaching adequate conformational sampling, overly simplistic treatment of electronic polarization, and deficiencies in force field parameterization still must be addressed. Active research in these areas is ongoing, knowledge of the rapid development of current approaches and their elevated performance compared to studies completed only a few years ago inspires confidence that the grand challenge of accurate binding free energy prediction is achievable in the near future.

## References

[B1] Aghaee E. Ghodrati M. Ghasemi J. B. (2021). In Silico exploration of Novel Protease Inhibitors against Coronavirus 2019 (COVID-19). Inform. Med. Unlocked 23, 100516. 10.1016/j.imu.2021.100516 33457495 PMC7801185

[B2] Ahmad K. Balaramnavar V. M. Chaturvedi N. Khan S. Haque S. Lee Y. H. (2019). Targeting Caspase 8: Using Structural and Ligand-Based Approaches to Identify Potential Leads for the Treatment of Multi-Neurodegenerative Diseases. Molecules 24. 10.3390/molecules24091827 PMC6539313 31083628

[B3] Al-Madhagi W. M. Hashim N. M. Awadh Ali N. A. Taha H. Alhadi A. A. Abdullah A. A. (2019). Bioassay-Guided Isolation and In Silico Study of Antibacterial Compounds from Petroleum Ether Extract of Peperomia Blanda (Jacq.) Kunth. J. Chem. Inf. Model. 59, 1858–1872. 10.1021/acs.jcim.8b00969 31117526

[B4] Aldeghi M. Bodkin M. J. Knapp S. Biggin P. C. (2017). Statistical Analysis on the Performance of Molecular Mechanics Poisson-Boltzmann Surface Area versus Absolute Binding Free Energy Calculations: Bromodomains as a Case Study. J. Chem. Inf. Model. 57, 2203–2221. 10.1021/acs.jcim.7b00347 28786670 PMC5615372

[B5] Aldeghi M. Gapsys V. De Groot B. L. (2018). Accurate Estimation of Ligand Binding Affinity Changes upon Protein Mutation. ACS Cent. Sci. 4, 1708–1718. 10.1021/acscentsci.8b00717 30648154 PMC6311686

[B6] Aleksandrov A. Lin F.-Y. Roux B. Mackerell A. D. Jr. (2018). Combining the Polarizable Drude Force Field with a Continuum Electrostatic Poisson-Boltzmann Implicit Solvation Model. J. Comput. Chem. 39, 1707–1719. 10.1002/jcc.25345 29737546 PMC6108925

[B7] Aleksandrov A. Roux B. Mackerell A. D. Jr. (2020). pKa Calculations with the Polarizable Drude Force Field and Poisson-Boltzmann Solvation Model. J. Chem. Theor. Comput. 16, 4655–4668. 10.1021/acs.jctc.0c00111 PMC7428141 32464053

[B8] Almlöf M. Brandsdal B. O. Åqvist J. (2004). Binding Affinity Prediction with Different Force fields: Examination of the Linear Interaction Energy Method. J. Comput. Chem. 25, 1242–1254. 10.1002/jcc.20047 15139037

[B9] Almlöf M. Carlsson J. Åqvist J. (2007). Improving the Accuracy of the Linear Interaction Energy Method for Solvation Free Energies. J. Chem. Theor. Comput. 3, 2162–2175. 10.1021/ct700106b 26636209

[B10] Amezcua M. El Khoury L. Mobley D. L. (2021). SAMPL7 Host-Guest Challenge Overview: Assessing the Reliability of Polarizable and Non-polarizable Methods for Binding Free Energy Calculations. J. Comput. Aided Mol. Des. 10.1007/s10822-020-00363-5 PMC8121194 33392951

[B11] Anwar J. Heyes D. M. (2005). Robust and Accurate Method for Free-Energy Calculation of Charged Molecular Systems. J. Chem. Phys. 122, 224117. 10.1063/1.1924449 15974661

[B12] Åqvist J. Hansson T. (1996). On the Validity of Electrostatic Linear Response in Polar Solvents. J. Phys. Chem. 100, 9512–9521. 10.1021/jp953640a

[B13] Åqvist J. Luzhkov V. B. Brandsdal B. O. (2002). Ligand Binding Affinities from MD Simulations. Acc. Chem. Res. 35, 358–365. 10.1021/ar010014p 12069620

[B14] Aqvist J. Marelius J. (2001). The Linear Interaction Energy Method for Predicting Ligand Binding Free Energies. Cchts 4, 613–626. 10.2174/1386207013330661 11812258

[B15] Åqvist J. Medina C. Samuelsson J.-E. (1994). A New Method for Predicting Binding Affinity in Computer-Aided Drug Design. Protein Eng. Des. Sel 7, 385–391. 10.1093/protein/7.3.385 8177887

[B16] Azhagiya Singam E. R. Tachachartvanich P. La Merrill M. A. Smith M. T. Durkin K. A. (2019). Structural Dynamics of Agonist and Antagonist Binding to the Androgen Receptor. J. Phys. Chem. B 123, 7657–7666. 10.1021/acs.jpcb.9b05654 31431014 PMC6742532

[B17] Bashford D. Karplus M. (1990). pKa's of Ionizable Groups in Proteins: Atomic Detail from a Continuum Electrostatic Model. Biochemistry 29, 10219–10225. 10.1021/bi00496a010 2271649

[B18] Bennett C. H. (1976). Efficient Estimation of Free Energy Differences from Monte Carlo Data. J. Comput. Phys. 22, 245–268. 10.1016/0021-9991(76)90078-4

[B19] Berishvili V. P. Kuimov A. N. Voronkov A. E. Radchenko E. V. Kumar P. Choonara Y. E. (2020). Discovery of Novel Tankyrase Inhibitors through Molecular Docking-Based Virtual Screening and Molecular Dynamics Simulation Studies. Molecules 25. 10.3390/molecules25143171 PMC7397142 32664504

[B20] Berishvili V. P. Perkin V. O. Voronkov A. E. Radchenko E. V. Syed R. Venkata Ramana Reddy C. (2019). Time-Domain Analysis of Molecular Dynamics Trajectories Using Deep Neural Networks: Application to Activity Ranking of Tankyrase Inhibitors. J. Chem. Inf. Model. 59, 3519–3532. 10.1021/acs.jcim.9b00135 31276400

[B21] Beutler T. C. Mark A. E. Van Schaik R. C. Gerber P. R. Van Gunsteren W. F. (1994). Avoiding Singularities and Numerical Instabilities in Free Energy Calculations Based on Molecular Simulations. Chem. Phys. Lett. 222, 529–539. 10.1016/0009-2614(94)00397-1

[B22] Bhardwaj V. K. Singh R. Das P. Purohit R. (2021). Evaluation of Acridinedione Analogs as Potential SARS-CoV-2 Main Protease Inhibitors and Their Comparison with Repurposed Anti-viral Drugs. Comput. Biol. Med. 128, 104117. 10.1016/j.compbiomed.2020.104117 33217661 PMC7659809

[B23] Bhati A. P. Wan S. Coveney P. V. (2019). Ensemble-Based Replica Exchange Alchemical Free Energy Methods: The Effect of Protein Mutations on Inhibitor Binding. J. Chem. Theor. Comput. 15, 1265–1277. 10.1021/acs.jctc.8b01118 PMC6447239 30592603

[B24] Boresch S. Tettinger F. Leitgeb M. Karplus M. (2003). Absolute Binding Free Energies: A Quantitative Approach for Their Calculation. J. Phys. Chem. B 107, 9535–9551. 10.1021/jp0217839

[B25] Bosken Y. K. Cholko T. Lou Y. C. Wu K. P. Chang C. A. (2020). Insights into Dynamics of Inhibitor and Ubiquitin-like Protein Binding in SARS-CoV-2 Papain-like Protease. Front. Mol. Biosci. 7, 174. 10.3389/fmolb.2020.00174 32850963 PMC7417481

[B26] Botelho F. D. Dos Santos M. C. Goncalves A. D. S. Kuca K. Valis M. Laplante S. R. (2020). Ligand-Based Virtual Screening, Molecular Docking, Molecular Dynamics, and MM-PBSA Calculations towards the Identification of Potential Novel Ricin Inhibitors. Toxins (Basel) 12. 10.3390/toxins12120746 PMC7761309 33256167

[B27] Botello-Smith W. M. Liu X. Cai Q. Li Z. Zhao H. Luo R. (2012). Numerical Poisson-Boltzmann Model for Continuum Membrane Systems. Chem. Phys. Lett. 10.1016/j.cplett.2012.10.081 PMC3579545 23439886

[B28] Bruckner S. Boresch S. (2011a). Efficiency of Alchemical Free Energy Simulations. I. A Practical Comparison of the Exponential Formula, Thermodynamic Integration, and Bennett's Acceptance Ratio Method. J. Comput. Chem. 32, 1303–1319. 10.1002/jcc.21713 21425288

[B29] Bruckner S. Boresch S. (2011b). Efficiency of Alchemical Free Energy Simulations. II. Improvements for Thermodynamic Integration. J. Comput. Chem. 32, 1320–1333. 10.1002/jcc.21712 21425289

[B30] Cabeza De Vaca I. Qian Y. Vilseck J. Z. Tirado-Rives J. Jorgensen W. L. (2018). Enhanced Monte Carlo Methods for Modeling Proteins Including Computation of Absolute Free Energies of Binding. J. Chem. Theor. Comput. 14, 3279–3288. 10.1021/acs.jctc.8b00031 PMC6311413 29708338

[B31] Cai Q. Hsieh M.-J. Wang J. Luo R. (2010). Performance of Nonlinear Finite-Difference Poisson−Boltzmann Solvers. J. Chem. Theor. Comput. 6, 203–211. 10.1021/ct900381r PMC3979552 24723843

[B32] Cai Q. Wang J. Zhao H.-K. Luo R. (2009). On Removal of Charge Singularity in Poisson-Boltzmann Equation. J. Chem. Phys. 130. 10.1063/1.3099708 19368474

[B33] Cai Q. Ye X. Wang J. Luo R. (2011). On-the-Fly Numerical Surface Integration for Finite-Difference Poisson-Boltzmann Methods. J. Chem. Theor. Comput. 7, 3608–3619. 10.1021/ct200389p PMC3998210 24772042

[B34] Case D. A. Cheatham T. E. 3rd Darden T. Gohlke H. Luo R. Merz K. M. Jr. (2005). The Amber Biomolecular Simulation Programs. J. Comput. Chem. 26, 1668–1688. 10.1002/jcc.20290 16200636 PMC1989667

[B35] Case D. A. Ben-Shalom I. Y. Brozell S. R. Cerutti D. S. Cheatham T. E. Iii (2020). AMBER 2020. San Francisco: University of California.

[B36] Chakravorty A. Gallicchio E. Alexov E. (2019). A Grid‐based Algorithm in Conjunction with a Gaussian‐based Model of Atoms for Describing Molecular Geometry. J. Comput. Chem. 40, 1290–1304. 10.1002/jcc.25786 30698861 PMC6506848

[B37] Chang C.-e. A. Chen W. Gilson M. K. (2007). Ligand Configurational Entropy and Protein Binding. Proc. Natl. Acad. Sci. 104, 1534–1539. 10.1073/pnas.0610494104 17242351 PMC1780070

[B38] Chattaraj K. G. Paul S. (2020). Investigation on the Mechanisms of Synchronous Interaction of K3Cit with Melamine and Uric Acid that Avoids the Formation of Large Clusters. J. Chem. Inf. Model. 60, 4827–4844. 10.1021/acs.jcim.0c00384 32786693

[B39] Cheatham T. E. 3rd Srinivasan J. Case D. A. Kollman P. A. (1998). Molecular Dynamics and Continuum Solvent Studies of the Stability of polyG-polyC and polyA-polyT DNA Duplexes in Solution. J. Biomol. Struct. Dyn. 16, 265–280. 10.1080/07391102.1998.10508245 9833666

[B40] Chen B. Fountain G. Sullivan H.-J. Paradis N. Wu C. (2020a). To Probe the Binding Pathway of a Selective Compound (D089-0563) to C-MYC Pu24 G-Quadruplex Using Free Ligand Binding Simulations and Markov State Model Analysis. Phys. Chem. Chem. Phys. 22, 22567–22583. 10.1039/d0cp03863f 33000836

[B41] Chen F. Liu H. Sun H. Pan P. Li Y. Li D. (2016). Assessing the Performance of the MM/PBSA and MM/GBSA Methods. 6. Capability to Predict Protein-Protein Binding Free Energies and Re-rank Binding Poses Generated by Protein-Protein Docking. Phys. Chem. Chem. Phys. 18, 22129–22139. 10.1039/c6cp03670h 27444142

[B42] Chen F. Sun H. Wang J. Zhu F. Liu H. Wang Z. (2018a). Assessing the Performance of MM/PBSA and MM/GBSA Methods. 8. Predicting Binding Free Energies and Poses of Protein-RNA Complexes. RNA 24, 1183–1194. 10.1261/rna.065896.118 29930024 PMC6097651

[B43] Chen H. Maia J. D. C. Radak B. K. Hardy D. J. Cai W. Chipot C. (2020b). Boosting Free-Energy Perturbation Calculations with GPU-Accelerated NAMD. J. Chem. Inf. Model. 60, 5301–5307. 10.1021/acs.jcim.0c00745 32805108 PMC7686227

[B44] Chen J. Wang X. Pang L. Zhang J. Z. H. Zhu T. (2019). Effect of Mutations on Binding of Ligands to Guanine Riboswitch Probed by Free Energy Perturbation and Molecular Dynamics Simulations. Nucleic Acids Res. 47, 6618–6631. 10.1093/nar/gkz499 31173143 PMC6649850

[B45] Chen W. Chang C.-E. Gilson M. K. (2004). Calculation of Cyclodextrin Binding Affinities: Energy, Entropy, and Implications for Drug Design. Biophysical J. 87, 3035–3049. 10.1529/biophysj.104.049494 PMC1304776 15339804

[B46] Chen W. Deng Y. Russell E. Wu Y. Abel R. Wang L. (2018b). Accurate Calculation of Relative Binding Free Energies between Ligands with Different Net Charges. J. Chem. Theor. Comput. 14, 6346–6358. 10.1021/acs.jctc.8b00825 30375870

[B47] Chen Z. Wang H. Yang L. Jiang S. Wei D. (2021). Significantly Enhancing the Stereoselectivity of a Regioselective Nitrilase for the Production of (S)-3-cyano-5-methylhexanoic Acid Using an MM/PBSA Method. Chem. Commun. 57, 931–934. 10.1039/d0cc07106d 33398309

[B48] Chi L. A. Vargas M. C. (2020). In Silico design of Peptides as Potential Ligands to Resistin. J. Mol. Model. 26, 101. 10.1007/s00894-020-4338-3 32297015

[B49] Chodera J. D. (2016). A Simple Method for Automated Equilibration Detection in Molecular Simulations. J. Chem. Theor. Comput. 12, 1799–1805. 10.1021/acs.jctc.5b00784 PMC4945107 26771390

[B50] Chowdhury K. H. Chowdhury M. R. Mahmud S. Tareq A. M. Hanif N. B. Banu N. (2020). Drug Repurposing Approach against Novel Coronavirus Disease (COVID-19) through Virtual Screening Targeting SARS-CoV-2 Main Protease. Biology (Basel) 10. 10.3390/biology10010002 PMC7822464 33374717

[B51] Cooper C. D. (2019). A Boundary‐Integral Approach for the Poisson-Boltzmann Equation with Polarizable Force Fields. J. Comput. Chem. 40, 1680–1692. 10.1002/jcc.25820 30889283

[B52] Corey R. A. Vickery O. N. Sansom M. S. P. Stansfeld P. J. (2019). Insights into Membrane Protein-Lipid Interactions from Free Energy Calculations. J. Chem. Theor. Comput. 15, 5727–5736. 10.1021/acs.jctc.9b00548 PMC6785801 31476127

[B53] Cournia Z. Allen B. Sherman W. (2017). Relative Binding Free Energy Calculations in Drug Discovery: Recent Advances and Practical Considerations. J. Chem. Inf. Model. 57, 2911–2937. 10.1021/acs.jcim.7b00564 29243483

[B54] Cruz J. Wickstrom L. Yang D. Gallicchio E. Deng N. (2020). Combining Alchemical Transformation with a Physical Pathway to Accelerate Absolute Binding Free Energy Calculations of Charged Ligands to Enclosed Binding Sites. J. Chem. Theor. Comput. 16, 2803–2813. 10.1021/acs.jctc.9b01119 PMC7269639 32101691

[B55] Das B. K. Chakraborty D. (2020). Epitope-Based Potential Vaccine Candidate for Humoral and Cell-Mediated Immunity to Combat Severe Acute Respiratory Syndrome Coronavirus 2 Pandemic. J. Phys. Chem. Lett. 11, 9920–9930. 10.1021/acs.jpclett.0c02846 33174418

[B56] Davis M. E. Mccammon J. A. (1990). Electrostatics in Biomolecular Structure and Dynamics. Chem. Rev. 90, 509–521. 10.1021/cr00101a005

[B57] De Oliveira C. Yu H. S. Chen W. Abel R. Wang L. (2019). Rigorous Free Energy Perturbation Approach to Estimating Relative Binding Affinities between Ligands with Multiple Protonation and Tautomeric States. J. Chem. Theor. Comput. 15, 424–435. 10.1021/acs.jctc.8b00826 30537823

[B58] De Ruiter A. Boresch S. Oostenbrink C. (2013). Comparison of Thermodynamic Integration and Bennett Acceptance Ratio for Calculating Relative Protein-Ligand Binding Free Energies. J. Comput. Chem. 34, 1024–1034. 10.1002/jcc.23229 23335287

[B59] Deng N. Cui D. Zhang B. W. Xia J. Cruz J. Levy R. (2018). Comparing Alchemical and Physical Pathway Methods for Computing the Absolute Binding Free Energy of Charged Ligands. Phys. Chem. Chem. Phys. 20, 17081–17092. 10.1039/c8cp01524d 29896599 PMC6061996

[B60] Deng N. (2019). Using Molecular Dynamics Free Energy Simulation to Compute Binding Affinities of DNA G-Quadruplex Ligands. Methods Mol. Biol. 2035, 177–199. 10.1007/978-1-4939-9666-7_10 31444750

[B61] Deng N. Xia J. Wickstrom L. Lin C. Wang K. He P. (2019). Ligand Selectivity in the Recognition of Protoberberine Alkaloids by Hybrid-2 Human Telomeric G-Quadruplex: Binding Free Energy Calculation, Fluorescence Binding, and NMR Experiments. Molecules 24. 10.3390/molecules24081574 PMC6515380 31010072

[B62] Dey D. Borkotoky S. Banerjee M. (2020). In Silico identification of Tretinoin as a SARS-CoV-2 Envelope (E) Protein Ion Channel Inhibitor. Comput. Biol. Med. 127, 104063. 10.1016/j.compbiomed.2020.104063 33126128 PMC7574788

[B63] Di Sotto A. Irannejad H. Eufemi M. Mancinelli R. Abete L. Mammola C. L. (2020). Potentiation of Low-Dose Doxorubicin Cytotoxicity by Affecting P-Glycoprotein through Caryophyllane Sesquiterpenes in HepG2 Cells: an *In Vitro* and In Silico Study. Int. J. Mol. Sci. 21. 10.3390/ijms21020633 PMC7014471 31963614

[B64] Dimasi J. A. Grabowski H. G. Hansen R. W. (2016). Innovation in the Pharmaceutical Industry: New Estimates of R&D Costs. J. Health Econ. 47, 20–33. 10.1016/j.jhealeco.2016.01.012 26928437

[B65] Ding X. Zhang B. (2021). DeepBAR: A Fast and Exact Method for Binding Free Energy Computation. J. Phys. Chem. Lett. 12, 2509–2515. 10.1021/acs.jpclett.1c00189 33719449 PMC8030779

[B66] Dixit S. B. Chipot C. (2001). Can Absolute Free Energies of Association Be Estimated from Molecular Mechanical Simulations? the Biotin−Streptavidin System Revisited. J. Phys. Chem. A. 105, 9795–9799. 10.1021/jp011878v

[B67] Edinger S. R. Cortis C. Shenkin P. S. Friesner R. A. (1997). Solvation Free Energies of Peptides: Comparison of Approximate Continuum Solvation Models with Accurate Solution of the Poisson−Boltzmann Equation. J. Phys. Chem. B 101, 1190–1197. 10.1021/jp962156k

[B68] Eisenberg D. Mclachlan A. D. (1986). Solvation Energy in Protein Folding and Binding. Nature 319, 199–203. 10.1038/319199a0 3945310

[B69] Ekimoto T. Yamane T. Ikeguchi M. (2018). Elimination of Finite-Size Effects on Binding Free Energies via the Warp-Drive Method. J. Chem. Theor. Comput. 14, 6544–6559. 10.1021/acs.jctc.8b00280 30404450

[B70] El Hassab M. A. Shoun A. A. Al-Rashood S. T. Al-Warhi T. Eldehna W. M. (2020). Identification of a New Potential SARS-COV-2 RNA-dependent RNA Polymerase Inhibitor via Combining Fragment-Based Drug Design, Docking, Molecular Dynamics, and MM-PBSA Calculations. Front. Chem. 8, 584894. 10.3389/fchem.2020.584894 33195080 PMC7662682

[B71] Faulkner C. Plant D. F. De Leeuw N. H. (2019). Modulation of the Gloeobacter Violaceus Ion Channel by Fentanyl: A Molecular Dynamics Study. Biochemistry 58, 4804–4808. 10.1021/acs.biochem.9b00881 31718178

[B72] Fiorentini R. Kremer K. Potestio R. (2020). Ligand‐protein Interactions in Lysozyme Investigated through a Dual‐resolution Model. Proteins 88, 1351–1360. 10.1002/prot.25954 32525263 PMC7497117

[B73] Fu H. Gumbart J. C. Chen H. Shao X. Cai W. Chipot C. (2018). BFEE: A User-Friendly Graphical Interface Facilitating Absolute Binding Free-Energy Calculations. J. Chem. Inf. Model. 58, 556–560. 10.1021/acs.jcim.7b00695 29405709 PMC5869121

[B74] Fu Y. Liu Y. X. Yi K. H. Li M. Q. Li J. Z. Ye F. (2019). Quantitative Structure Activity Relationship Studies and Molecular Dynamics Simulations of 2-(Aryloxyacetyl)cyclohexane-1,3-Diones Derivatives as 4-Hydroxyphenylpyruvate Dioxygenase Inhibitors. Front. Chem. 7, 556. 10.3389/fchem.2019.00556 31482084 PMC6710436

[B75] Gan W. Roux B. (2009). Binding Specificity of SH2 Domains: Insight from Free Energy Simulations. Proteins 74, 996–1007. 10.1002/prot.22209 18767163 PMC2635922

[B76] Genheden S. Ryde U. (2015). The MM/PBSA and MM/GBSA Methods to Estimate Ligand-Binding Affinities. Expert Opin. Drug Discov. 10, 449–461. 10.1517/17460441.2015.1032936 25835573 PMC4487606

[B77] Gharouni M. Mosaddeghi H. Mehrzad J. Es-Haghi A. Motavalizadehkakhky A. (2021). In Silico profiling and Structural Insights of Zinc Metal Ion on O6-Methylguanine Methyl Transferase and its Interactions Using Molecular Dynamics Approach. J. Mol. Model. 27, 40. 10.1007/s00894-020-04631-x 33454889

[B78] Gheibi N. Ghorbani M. Shariatifar H. Farasat A. (2019). In Silico assessment of Human Calprotectin Subunits (S100A8/A9) in Presence of Sodium and Calcium Ions Using Molecular Dynamics Simulation Approach. PLoS One 14, e0224095. 10.1371/journal.pone.0224095 31622441 PMC6797115

[B79] Giese T. J. York D. M. (2018). A GPU-Accelerated Parameter Interpolation Thermodynamic Integration Free Energy Method. J. Chem. Theor. Comput. 14, 1564–1582. 10.1021/acs.jctc.7b01175 PMC5849537 29357243

[B80] Gilson M. K. Given J. A. Bush B. L. Mccammon J. A. (1997). The Statistical-Thermodynamic Basis for Computation of Binding Affinities: a Critical Review. Biophysical J. 72, 1047–1069. 10.1016/s0006-3495(97)78756-3 PMC1184492 9138555

[B81] Gilson M. K. (1995). Theory of Electrostatic Interactions in Macromolecules. Curr. Opin. Struct. Biol. 5, 216–223. 10.1016/0959-440x(95)80079-4 7648324

[B82] Gohlke H. Case D. A. (2004). Converging Free Energy Estimates: MM-PB(GB)SA Studies on the Protein-Protein Complex Ras-Raf. J. Comput. Chem. 25, 238–250. 10.1002/jcc.10379 14648622

[B83] Granadino-Roldan J. M. Mey A. Perez Gonzalez J. J. Bosisio S. Rubio-Martinez J. Michel J. (2019). Effect of Set up Protocols on the Accuracy of Alchemical Free Energy Calculation over a Set of ACK1 Inhibitors. PLoS One 14, e0213217. 10.1371/journal.pone.0213217 30861030 PMC6413950

[B84] Greene D. A. Botello-Smith W. M. Follmer A. Xiao L. Lambros E. Luo R. (2016). Modeling Membrane Protein-Ligand Binding Interactions: The Human Purinergic Platelet Receptor. J. Phys. Chem. B 120, 12293–12304. 10.1021/acs.jpcb.6b09535 27934233 PMC5460638

[B85] Greene D. A. Po T. Pan J. Tabibian T. Luo R. (2018). Computational Analysis for the Rational Design of Anti-amyloid Beta (Aβ) Antibodies. J. Phys. Chem. B 122, 4521–4536. 10.1021/acs.jpcb.8b01837 29617557 PMC5927393

[B86] Greene D. A. Qi R. Nguyen R. Qiu T. Luo R. (2019). Heterogeneous Dielectric Implicit Membrane Model for the Calculation of MMPBSA Binding Free Energies. J. Chem. Inf. Model. 59, 3041–3056. 10.1021/acs.jcim.9b00363 31145610 PMC7197397

[B87] Gupta A. Rani C. Pant P. Vijayan V. Vikram N. Kaur P. (2020). Structure-Based Virtual Screening and Biochemical Validation to Discover a Potential Inhibitor of the SARS-CoV-2 Main Protease. ACS Omega 5, 33151–33161. 10.1021/acsomega.0c04808 33398250 PMC7754785

[B88] Gupta A. Zhou H.-X. (2020). Profiling SARS-CoV-2 Main Protease (MPRO) Binding to Repurposed Drugs Using Molecular Dynamics Simulations in Classical and Neural Network-Trained Force Fields. ACS Comb. Sci. 22, 826–832. 10.1021/acscombsci.0c00140 33119257 PMC7605330

[B89] Gupta S. Dasmahapatra A. K. (2020). Destabilization Potential of Phenolics on Aβ Fibrils: Mechanistic Insights from Molecular Dynamics Simulation. Phys. Chem. Chem. Phys. 22, 19643–19658. 10.1039/d0cp02459g 32830209

[B90] Gutiérrez-de-Terán H. Åqvist J. (2012). Linear Interaction Energy: Method and Applications in Drug Design. Methods Mol. Biol. 819, 305–323. 10.1007/978-1-61779-465-0_20 22183545

[B91] Han K. Hudson P. S. Jones M. R. Nishikawa N. Tofoleanu F. Brooks B. R. (2018). Prediction of CB[8] Host-Guest Binding Free Energies in SAMPL6 Using the Double-Decoupling Method. J. Comput. Aided Mol. Des. 32, 1059–1073. 10.1007/s10822-018-0144-8 30084077 PMC6347468

[B92] Hansson T. Marelius J. Åqvist J. (1998). Ligand Binding Affinity Prediction by Linear Interaction Energy Methods. J. Comput. Aided Mol. Des. 12, 27–35. 10.1023/a:1007930623000 9570087

[B93] Hao D. He X. Ji B. Zhang S. Wang J. (2020). How Well Does the Extended Linear Interaction Energy Method Perform in Accurate Binding Free Energy Calculations? J. Chem. Inf. Model. 60, 6624–6633. 10.1021/acs.jcim.0c00934 33213150

[B94] Harger M. Lee J. H. Walker B. Taliaferro J. M. Edupuganti R. Dalby K. N. (2019). Computational Insights into the Binding of IN17 Inhibitors to MELK. J. Mol. Model. 25, 151. 10.1007/s00894-019-4036-1 31069524 PMC7105934

[B95] Harger M. Li D. Wang Z. Dalby K. Lagardère L. Piquemal J. P. (2017). Tinker‐OpenMM: Absolute and Relative Alchemical Free Energies Using AMOEBA on GPUs. J. Comput. Chem. 38, 2047–2055. 10.1002/jcc.24853 28600826 PMC5539969

[B96] Hariono M. Choi S. B. Roslim R. F. Nawi M. S. Tan M. L. Kamarulzaman E. E. (2019). Thioguanine-based DENV-2 NS2B/NS3 Protease Inhibitors: Virtual Screening, Synthesis, Biological Evaluation and Molecular Modelling. PLoS One 14, e0210869. 10.1371/journal.pone.0210869 30677071 PMC6345492

[B97] Harish M. Kannan S. Puttagunta S. Pradhan M. R. Verma C. S. Venkatraman P. (2019). A Novel Determinant of PSMD9 PDZ Binding Guides the Evolution of the First Generation of Super Binding Peptides. Biochemistry 58, 3422–3433. 10.1021/acs.biochem.9b00308 31287951

[B98] Hazra T. Ahmed Ullah S. Wang S. Alexov E. Zhao S. (2019). A Super-gaussian Poisson-Boltzmann Model for Electrostatic Free Energy Calculation: Smooth Dielectric Distribution for Protein Cavities and in Both Water and Vacuum States. J. Math. Biol. 79, 631–672. 10.1007/s00285-019-01372-1 31030299 PMC9841320

[B99] He X. Liu S. Lee T.-S. Ji B. Man V. H. York D. M. (2020). Fast, Accurate, and Reliable Protocols for Routine Calculations of Protein-Ligand Binding Affinities in Drug Design Projects Using AMBER GPU-TI with ff14SB/GAFF. ACS Omega 5, 4611–4619. 10.1021/acsomega.9b04233 32175507 PMC7066661

[B100] He X. Man V. H. Ji B. Xie X.-Q. Wang J. (2019). Calculate Protein-Ligand Binding Affinities with the Extended Linear Interaction Energy Method: Application on the Cathepsin S Set in the D3R Grand Challenge 3. J. Comput. Aided Mol. Des. 33, 105–117. 10.1007/s10822-018-0162-6 30218199 PMC6608581

[B101] Head M. S. Given J. A. Gilson M. K. (1997). "Mining Minima": Direct Computation of Conformational Free Energy. J. Phys. Chem. A. 101, 1609–1618. 10.1021/jp963817g

[B102] Heinzelmann G. Gilson M. K. (2021). Automation of Absolute Protein-Ligand Binding Free Energy Calculations for Docking Refinement and Compound Evaluation. Sci. Rep. 11, 1116. 10.1038/s41598-020-80769-1 33441879 PMC7806944

[B103] Her Choong F. Keat Yap B. (2020). Cell-Penetrating Peptides: Correlation between Peptide-Lipid Interaction and Penetration Efficiency. Chemphyschem. 10.1002/cphc.202000873 33377300

[B104] Hitzenberger M. Zacharias M. (2019). Structural Modeling of γ-Secretase Aβn Complex Formation and Substrate Processing. ACS Chem. Neurosci. 10, 1826–1840. 10.1021/acschemneuro.8b00725 30638370

[B105] Honig B. Nicholls A. (1995). Classical Electrostatics in Biology and Chemistry. Science 268, 1144–1149. 10.1126/science.7761829 7761829

[B106] Hornak V. Simmerling C. (2004). Development of Softcore Potential Functions for Overcoming Steric Barriers in Molecular Dynamics Simulations. J. Mol. Graphics Model. 22, 405–413. 10.1016/j.jmgm.2003.12.007 15099836

[B107] Horoiwa S. Yokoi T. Masumoto S. Minami S. Ishizuka C. Kishikawa H. (2019). Structure-based Virtual Screening for Insect Ecdysone Receptor Ligands Using MM/PBSA. Bioorg. Med. Chem. 27, 1065–1075. 10.1016/j.bmc.2019.02.011 30770256

[B108] Hou Q. Zhang L. (2020). Biomimetic Design of Peptide Neutralizer of Ebola Virus with Molecular Simulation. Langmuir 36, 1813–1821. 10.1021/acs.langmuir.9b03832 31986884

[B109] Hou T. Wang J. Li Y. Wang W. (2011). Assessing the Performance of the MM/PBSA and MM/GBSA Methods. 1. The Accuracy of Binding Free Energy Calculations Based on Molecular Dynamics Simulations. J. Chem. Inf. Model. 51, 69–82. 10.1021/ci100275a 21117705 PMC3029230

[B110] Hsieh M.-J. Luo R. (2011). Exploring a Coarse-Grained Distributive Strategy for Finite-Difference Poisson-Boltzmann Calculations. J. Mol. Model. 17, 1985–1996. 10.1007/s00894-010-0904-4 21127924 PMC3143316

[B111] Hu G. Li H. Xu S. Wang J. (2020). Ligand Binding Mechanism and its Relationship with Conformational Changes in Adenine Riboswitch. Int. J. Mol. Sci. 21. 10.3390/ijms21061926 PMC7139962 32168940

[B112] Hu X. Contini A. (2019). Rescoring Virtual Screening Results with the MM-PBSA Methods: Beware of Internal Dielectric Constants. J. Chem. Inf. Model. 59, 2714–2728. 10.1021/acs.jcim.9b00095 31063686

[B113] Huai Z. Yang H. Li X. Sun Z. (2020). SAMPL7 TrimerTrip Host-Guest Binding Affinities from Extensive Alchemical and End-point Free Energy Calculations. J. Comput. Aided Mol. Des. 10.1007/s10822-020-00351-9 33037549

[B114] Huang K. Luo S. Cong Y. Zhong S. Zhang J. Z. H. Duan L. (2020). An Accurate Free Energy Estimator: Based on MM/PBSA Combined with Interaction Entropy for Protein-Ligand Binding Affinity. Nanoscale 12, 10737–10750. 10.1039/c9nr10638c 32388542

[B115] Hub J. S. De Groot B. L. Grubmüller H. Groenhof G. (2014). Quantifying Artifacts in Ewald Simulations of Inhomogeneous Systems with a Net Charge. J. Chem. Theor. Comput. 10, 381–390. 10.1021/ct400626b 26579917

[B116] Hünenberger P. H. Mccammon J. A. (1999). Effect of Artificial Periodicity in Simulations of Biomolecules under Ewald Boundary Conditions: a Continuum Electrostatics Study. Biophysical Chem. 78, 69–88. 10.1016/s0301-4622(99)00007-1 10343384

[B117] Isa D. M. Chin S. P. Chong W. L. Zain S. M. Rahman N. A. Lee V. S. (2019). Dynamics and Binding Interactions of Peptide Inhibitors of Dengue Virus Entry. J. Biol. Phys. 45, 63–76. 10.1007/s10867-018-9515-6 30680580 PMC6408556

[B118] Jafary F. Ganjalikhany M. R. Moradi A. Hemati M. Jafari S. (2019). Novel Peptide Inhibitors for Lactate Dehydrogenase A (LDHA): A Survey to Inhibit LDHA Activity via Disruption of Protein-Protein Interaction. Sci. Rep. 9, 4686. 10.1038/s41598-019-38854-7 30886157 PMC6423238

[B119] Jandova Z. Fast D. Setz M. Pechlaner M. Oostenbrink C. (2018). Saturation Mutagenesis by Efficient Free-Energy Calculation. J. Chem. Theor. Comput. 14, 894–904. 10.1021/acs.jctc.7b01099 PMC5813279 29262673

[B120] Jean-Charles A. Nicholls A. Sharp K. Honig B. Tempczyk A. Hendrickson T. F. (1991). Electrostatic Contributions to Solvation Energies: Comparison of Free Energy Perturbation and Continuum Calculations. J. Am. Chem. Soc. 113, 1454–1455. 10.1021/ja00004a079

[B121] Ji B. Liu S. He X. Man V. H. Xie X.-Q. Wang J. (2020). Prediction of the Binding Affinities and Selectivity for CB1 and CB2 Ligands Using Homology Modeling, Molecular Docking, Molecular Dynamics Simulations, and MM-PBSA Binding Free Energy Calculations. ACS Chem. Neurosci. 11, 1139–1158. 10.1021/acschemneuro.9b00696 32196303

[B122] Jiang S. J. Tsai P. I. Peng S. Y. Chang C. C. Chung Y. Tsao H. H. (2019). A Potential Peptide Derived from Cytokine Receptors Can Bind Proinflammatory Cytokines as a Therapeutic Strategy for Anti-inflammation. Sci. Rep. 9, 2317. 10.1038/s41598-018-36492-z 30783144 PMC6381106

[B123] Jiang W. (2019). Accelerating Convergence of Free Energy Computations with Hamiltonian Simulated Annealing of Solvent (HSAS). J. Chem. Theor. Comput. 15, 2179–2186. 10.1021/acs.jctc.8b01147 30821969

[B124] Jiang W. Thirman J. Jo S. Roux B. (2018). Reduced Free Energy Perturbation/Hamiltonian Replica Exchange Molecular Dynamics Method with Unbiased Alchemical Thermodynamic Axis. J. Phys. Chem. B 122, 9435–9442. 10.1021/acs.jpcb.8b03277 30253098 PMC6339808

[B125] Jing Z. Liu C. Qi R. Ren P. (2018). Many-body Effect Determines the Selectivity for Ca2+ and Mg2+ in Proteins. Proc. Natl. Acad. Sci. USA 115, E7495–E7501. 10.1073/pnas.1805049115 30038003 PMC6094099

[B126] Jukic M. Janezic D. Bren U. (2020). Ensemble Docking Coupled to Linear Interaction Energy Calculations for Identification of Coronavirus Main Protease (3CL(pro)) Non-covalent Small-Molecule Inhibitors. Molecules 25 10.3390/molecules25245808 PMC7763084 33316996

[B127] Jung S. W. Cho A. E. Yu W. (2018). Exploring the Ligand Efficacy of Cannabinoid Receptor 1 (CB1) Using Molecular Dynamics Simulations. Sci. Rep. 8, 13787. 10.1038/s41598-018-31749-z 30213978 PMC6137198

[B128] Kang H. Yang Z. Zhou R. (2018). Lanosterol Disrupts Aggregation of Human γD-Crystallin by Binding to the Hydrophobic Dimerization Interface. J. Am. Chem. Soc. 140, 8479–8486. 10.1021/jacs.8b03065 29916249

[B129] Kang N. Liu J. Zhao Y. (2019). Dissociation Mechanism of GDP from Cdc42 via DOCK9 Revealed by Molecular Dynamics Simulations. Proteins 87, 433–442. 10.1002/prot.25665 30714195

[B130] Kassem S. Ahmed M. El-Sheikh S. Barakat K. H. (2015). Entropy in Bimolecular Simulations: A Comprehensive Review of Atomic Fluctuations-Based Methods. J. Mol. Graphics Model. 62, 105–117. 10.1016/j.jmgm.2015.09.010 26407139

[B131] Kaushik S. Marques S. M. Khirsariya P. Paruch K. Libichova L. Brezovsky J. (2018). Impact of the Access Tunnel Engineering on Catalysis Is Strictly Ligand‐specific. FEBS J. 285, 1456–1476. 10.1111/febs.14418 29478278

[B132] Kawai R. Araki M. Yoshimura M. Kamiya N. Ono M. Saji H. (2018). Core Binding Site of a Thioflavin-T-Derived Imaging Probe on Amyloid β Fibrils Predicted by Computational Methods. ACS Chem. Neurosci. 9, 957–966. 10.1021/acschemneuro.7b00389 29381047

[B133] Khalak Y. Tresadern G. De Groot B. L. Gapsys V. (2020). Non-equilibrium Approach for Binding Free Energies in Cyclodextrins in SAMPL7: Force fields and Software. J. Comput. Aided Mol. Des. 10.1007/s10822-020-00359-1 PMC7862541 33230742

[B134] Kilburg D. Gallicchio E. (2018). Assessment of a Single Decoupling Alchemical Approach for the Calculation of the Absolute Binding Free Energies of Protein-Peptide Complexes. Front. Mol. Biosci. 5, 22. 10.3389/fmolb.2018.00022 29568737 PMC5852065

[B135] Kim S. Oshima H. Zhang H. Kern N. R. Re S. Lee J. (2020). CHARMM-GUI Free Energy Calculator for Absolute and Relative Ligand Solvation and Binding Free Energy Simulations. J. Chem. Theor. Comput. 16, 7207–7218. 10.1021/acs.jctc.0c00884 PMC7658063 33112150

[B136] King E. Qi R. Li H. Luo R. Aitchison E. (2021). Estimating the Roles of Protonation and Electronic Polarization in Absolute Binding Affinity Simulations. J. Chem. Theor. Comput. 10.1021/acs.jctc.0c01305 PMC8254375 33764050

[B137] Kirkwood J. G. A. B. J. (1967). Theory of Liquids. New York: Gordon & Breach Science.

[B138] Kirkwood J. G. (1935). Statistical Mechanics of Fluid Mixtures. J. Chem. Phys. 3, 300–313. 10.1063/1.1749657

[B139] Klimovich P. V. Shirts M. R. Mobley D. L. (2015). Guidelines for the Analysis of Free Energy Calculations. J. Comput. Aided Mol. Des. 29, 397–411. 10.1007/s10822-015-9840-9 25808134 PMC4420631

[B140] Kollman P. A. Massova I. Reyes C. Kuhn B. Huo S. Chong L. (2000). Calculating Structures and Free Energies of Complex Molecules: Combining Molecular Mechanics and Continuum Models. Acc. Chem. Res. 33, 889–897. 10.1021/ar000033j 11123888

[B141] König G. Glaser N. Schroeder B. Kubincová A. Hünenberger P. H. Riniker S. (2020). An Alternative to Conventional λ-Intermediate States in Alchemical Free Energy Calculations: λ-Enveloping Distribution Sampling. J. Chem. Inf. Model. 60, 5407–5423. 10.1021/acs.jcim.0c00520 32794763

[B142] Kuhn M. Firth-Clark S. Tosco P. Mey A. S. J. S. Mackey M. Michel J. (2020). Assessment of Binding Affinity via Alchemical Free-Energy Calculations. J. Chem. Inf. Model. 60, 3120–3130. 10.1021/acs.jcim.0c00165 32437145

[B143] Kumar V. Naumann M. Stein M. (2018). Computational Studies on the Inhibitor Selectivity of Human JAMM Deubiquitinylases Rpn11 and CSN5. Front. Chem. 6, 480. 10.3389/fchem.2018.00480 30356695 PMC6189316

[B144] Lagarias P. Barkan K. Tzortzini E. Stampelou M. Vrontaki E. Ladds G. (2019). Insights to the Binding of a Selective Adenosine A3 Receptor Antagonist Using Molecular Dynamic Simulations, MM-PBSA and MM-GBSA Free Energy Calculations, and Mutagenesis. J. Chem. Inf. Model. 59, 5183–5197. 10.1021/acs.jcim.9b00751 31725294

[B145] Lamazares E. Macleod-Carey D. Miranda F. P. Mena-Ulecia K. (2021). Theoretical Evaluation of Novel Thermolysin Inhibitors from Bacillus Thermoproteolyticus. Possible Antibacterial AgentsMolecules 26. 10.3390/molecules26020386 PMC7828527 33451037

[B146] Laudadio E. Cedraro N. Mangiaterra G. Citterio B. Mobbili G. Minnelli C. (2019). Natural Alkaloid Berberine Activity against *Pseudomonas aeruginosa* MexXY-Mediated Aminoglycoside Resistance: In Silico and *In Vitro* Studies. J. Nat. Prod. 82, 1935–1944. 10.1021/acs.jnatprod.9b00317 31274312

[B147] Laury M. L. Wang Z. Gordon A. S. Ponder J. W. (2018). Absolute Binding Free Energies for the SAMPL6 Cucurbit[8]uril Host-Guest challenge via the AMOEBA Polarizable Force Field. J. Comput. Aided Mol. Des. 32, 1087–1095. 10.1007/s10822-018-0147-5 30324303 PMC6240481

[B148] Lee A. Geng W. Zhao S. (2021). Regularization Methods for the Poisson-Boltzmann Equation: Comparison and Accuracy Recovery. J. Comput. Phys. 426. 10.1016/j.jcp.2020.109958

[B149] Lee M. S. Olson M. A. (2006). Calculation of Absolute Protein-Ligand Binding Affinity Using Path and Endpoint Approaches. Biophysical J. 90, 864–877. 10.1529/biophysj.105.071589 PMC1367111 16284269

[B150] Lee T.-S. Allen B. K. Giese T. J. Guo Z. Li P. Lin C. (2020a). Alchemical Binding Free Energy Calculations in AMBER20: Advances and Best Practices for Drug Discovery. J. Chem. Inf. Model. 60, 5595–5623. 10.1021/acs.jcim.0c00613 32936637 PMC7686026

[B151] Lee T.-S. Lin Z. Allen B. K. Lin C. Radak B. K. Tao Y. (2020b). Improved Alchemical Free Energy Calculations with Optimized Smoothstep Softcore Potentials. J. Chem. Theor. Comput. 16, 5512–5525. 10.1021/acs.jctc.0c00237 PMC7494069 32672455

[B152] Li Y. Cong Y. Feng G. Zhong S. Zhang J. Z. H. Sun H. (2018). The Impact of interior Dielectric Constant and Entropic Change on HIV-1 Complex Binding Free Energy Prediction. Struct. Dyn. 5, 064101. 10.1063/1.5058172 30868080 PMC6404944

[B153] Li Y. Nam K. (2020). Repulsive Soft-Core Potentials for Efficient Alchemical Free Energy Calculations. J. Chem. Theor. Comput. 16, 4776–4789. 10.1021/acs.jctc.0c00163 PMC8324312 32559374

[B154] Li Y. Netherland M. D. Zhang C. Hong H. Gong P. (2019a). In Silico identification of Genetic Mutations Conferring Resistance to Acetohydroxyacid Synthase Inhibitors: A Case Study of Kochia Scoparia. PLoS One 14, e0216116. 10.1371/journal.pone.0216116 31063467 PMC6504096

[B155] Li Z. Huang Y. Wu Y. Chen J. Wu D. Zhan C.-G. (2019b). Absolute Binding Free Energy Calculation and Design of a Subnanomolar Inhibitor of Phosphodiesterase-10. J. Med. Chem. 62, 2099–2111. 10.1021/acs.jmedchem.8b01763 30689375

[B156] Li Z. Li X. Huang Y.-Y. Wu Y. Liu R. Zhou L. (2020). Identify Potent SARS-CoV-2 Main Protease Inhibitors via Accelerated Free Energy Perturbation-Based Virtual Screening of Existing Drugs. Proc. Natl. Acad. Sci. USA 117, 27381–27387. 10.1073/pnas.2010470117 33051297 PMC7959488

[B157] Lin Y.-L. Aleksandrov A. Simonson T. Roux B. (2014). An Overview of Electrostatic Free Energy Computations for Solutions and Proteins. J. Chem. Theor. Comput. 10, 2690–2709. 10.1021/ct500195p 26586504

[B158] Liu X. Wang C. Wang J. Li Z. Zhao H. Luo R. (2013). Exploring a Charge-central Strategy in the Solution of Poisson's Equation for Biomolecular Applications. Phys. Chem. Chem. Phys. 10.1039/c2cp41894k PMC3518737 23147243

[B159] Loeffler H. H. Bosisio S. Duarte Ramos Matos G. Suh D. Roux B. Mobley D. L. (2018). Reproducibility of Free Energy Calculations across Different Molecular Simulation Software Packages. J. Chem. Theor. Comput. 14, 5567–5582. 10.1021/acs.jctc.8b00544 30289712

[B160] Lu N. Singh J. K. Kofke D. A. (2003). Appropriate Methods to Combine Forward and Reverse Free-Energy Perturbation Averages. J. Chem. Phys. 118, 2977–2984. 10.1063/1.1537241

[B161] Lu Q. Luo R. (2003). A Poisson-Boltzmann Dynamics Method with Nonperiodic Boundary Condition. J. Chem. Phys. 119, 11035–11047. 10.1063/1.1622376

[B162] Luo R. David L. Gilson M. K. (2002). Accelerated Poisson-Boltzmann Calculations for Static and Dynamic Systems. J. Comput. Chem. 23, 1244–1253. 10.1002/jcc.10120 12210150

[B163] Luo R. Gilson M. K. (2000). Synthetic Adenine Receptors: Direct Calculation of Binding Affinity and Entropy. J. Am. Chem. Soc. 122, 2934–2937. 10.1021/ja994034m

[B164] Luo R. Head M. S. Given J. A. Gilson M. K. (1999). Nucleic Acid Base-Pairing and N-Methylacetamide Self-Association in Chloroform: Affinity and Conformation. Biophysical Chem. 78, 183–193. 10.1016/s0301-4622(98)00229-4 10343387

[B165] Luo R. Moult J. Gilson M. K. (1997). Dielectric Screening Treatment of Electrostatic Solvation. J. Phys. Chem. B 101, 11226–11236. 10.1021/jp9724838

[B166] Macchiagodena M. Pagliai M. Andreini C. Rosato A. Procacci P. (2020a). Upgraded AMBER Force Field for Zinc-Binding Residues and Ligands for Predicting Structural Properties and Binding Affinities in Zinc-Proteins. ACS Omega 5, 15301–15310. 10.1021/acsomega.0c01337 32637803 PMC7331063

[B167] Macchiagodena M. Pagliai M. Andreini C. Rosato A. Procacci P. (2019). Upgrading and Validation of the AMBER Force Field for Histidine and Cysteine Zinc(II)-Binding Residues in Sites with Four Protein Ligands. J. Chem. Inf. Model. 59, 3803–3816. 10.1021/acs.jcim.9b00407 31385702

[B168] Macchiagodena M. Pagliai M. Karrenbrock M. Guarnieri G. Iannone F. Procacci P. (2020b). Virtual Double-System Single-Box: A Nonequilibrium Alchemical Technique for Absolute Binding Free Energy Calculations: Application to Ligands of the SARS-CoV-2 Main Protease. J. Chem. Theor. Comput. 16, 7160–7172. 10.1021/acs.jctc.0c00634 PMC8015232 33090785

[B169] Mann G. Hermans J. (2000). Modeling Protein-Small Molecule Interactions: Structure and Thermodynamics of noble Gases Binding in a Cavity in Mutant Phage T4 Lysozyme L99A. J. Mol. Biol. 302, 979–989. 10.1006/jmbi.2000.4064 10993736

[B170] Mardis K. L. Luo R. Gilson M. K. (2001). Interpreting Trends in the Binding of Cyclic Ureas to HIV-1 Protease. J. Mol. Biol. 309, 507–517. 10.1006/jmbi.2001.4668 11371168

[B171] Mariadasse R. Choubey S. K. Jeyakanthan J. (2020). Insights into Exogenous Tryptophan-Mediated Allosteric Communication and Helical Transition of TRP Protein for Transcription Regulation. J. Chem. Inf. Model. 60, 175–191. 10.1021/acs.jcim.9b00755 31742398

[B172] Martin W. R. Lightstone F. C. Cheng F. (2020). In Silico Insights into Protein-Protein Interaction Disruptive Mutations in the PCSK9-LDLR Complex. Int. J. Mol. Sci. 21. 10.3390/ijms21051550 PMC7084799 32106405

[B173] Martins L. C. Torres P. H. M. De Oliveira R. B. Pascutti P. G. Cino E. A. Ferreira R. S. (2018). Investigation of the Binding Mode of a Novel Cruzain Inhibitor by Docking, Molecular Dynamics, Ab Initio and MM/PBSA Calculations. J. Comput. Aided Mol. Des. 32, 591–605. 10.1007/s10822-018-0112-3 29564808

[B174] Matsunaga Y. Yamane T. Terada T. Moritsugu K. Fujisaki H. Murakami S. (2018). Energetics and Conformational Pathways of Functional Rotation in the Multidrug Transporter AcrB. Elife 7. 10.7554/elife.31715 PMC5839741 29506651

[B175] Menzer W. M. Li C. Sun W. Xie B. Minh D. D. L. (2018). Simple Entropy Terms for End-Point Binding Free Energy Calculations. J. Chem. Theor. Comput. 14, 6035–6049. 10.1021/acs.jctc.8b00418 PMC6440217 30296084

[B176] Milenković D. A. Dimić D. S. Avdović E. H. Marković Z. S. (2020). Several Coumarin Derivatives and Their Pd(ii) Complexes as Potential Inhibitors of the Main Protease of SARS-CoV-2, an In Silico Approach. RSC Adv. 10, 35099–35108. 10.1039/d0ra07062a PMC9056878 35515669

[B177] Miller B. R. 3rd Mcgee T. D. Jr. Swails J. M. Homeyer N. Gohlke H. Roitberg A. E. (2012). MMPBSA.py: An Efficient Program for End-State Free Energy Calculations. J. Chem. Theor. Comput. 8, 3314–3321. 10.1021/ct300418h 26605738

[B178] Minh D. D. L. (2020). Alchemical Grid Dock (AlGDock): Binding Free Energy Calculations between Flexible Ligands and Rigid Receptors. J. Comput. Chem. 41, 715–730. 10.1002/jcc.26036 31397498 PMC7263302

[B179] Minuesa G. Albanese S. K. Xie W. Kazansky Y. Worroll D. Chow A. (2019). Small-molecule Targeting of MUSASHI RNA-Binding Activity in Acute Myeloid Leukemia. Nat. Commun. 10, 2691. 10.1038/s41467-019-10523-3 31217428 PMC6584500

[B180] Mishra S. K. Koča J. (2018). Assessing the Performance of MM/PBSA, MM/GBSA, and QM-MM/GBSA Approaches on Protein/Carbohydrate Complexes: Effect of Implicit Solvent Models, QM Methods, and Entropic Contributions. J. Phys. Chem. B 122, 8113–8121. 10.1021/acs.jpcb.8b03655 30084252

[B181] Mobley D. L. Chodera J. D. Dill K. A. (2006). On the Use of Orientational Restraints and Symmetry Corrections in Alchemical Free Energy Calculations. J. Chem. Phys. 125, 084902. 10.1063/1.2221683 16965052 PMC3583553

[B182] Mobley D. L. Dill K. A. (2009). Binding of Small-Molecule Ligands to Proteins: "What You See" Is Not Always "What You Get". Structure 17, 489–498. 10.1016/j.str.2009.02.010 19368882 PMC2756098

[B183] Mobley D. L. Graves A. P. Chodera J. D. Mcreynolds A. C. Shoichet B. K. Dill K. A. (2007). Predicting Absolute Ligand Binding Free Energies to a Simple Model Site. J. Mol. Biol. 371, 1118–1134. 10.1016/j.jmb.2007.06.002 17599350 PMC2104542

[B184] Moesgaard L. Reinholdt P. Wüstner D. Kongsted J. (2020). Modeling the Sterol-Binding Domain of Aster-A Provides Insight into its Multiligand Specificity. J. Chem. Inf. Model. 60, 2268–2281. 10.1021/acs.jcim.0c00086 32233488

[B185] Moghaddam S. Yang C. Rekharsky M. Ko Y. H. Kim K. Inoue Y. (2011). New Ultrahigh Affinity Host−Guest Complexes of Cucurbit[7]uril with Bicyclo[2.2.2]octane and Adamantane Guests: Thermodynamic Analysis and Evaluation of M2 Affinity Calculations. J. Am. Chem. Soc. 133, 3570–3581. 10.1021/ja109904u 21341773 PMC3065999

[B186] Montalvo-Acosta J. J. Pacak P. Gomes D. E. B. Cecchini M. (2018). A Linear Interaction Energy Model for Cavitand Host-Guest Binding Affinities. J. Phys. Chem. B 122, 6810–6814. 10.1021/acs.jpcb.8b03245 29863347

[B187] Mottram L. Liu J. Chavan S. Tobias J. Svennerholm A. M. Holgersson J. (2018). Glyco-engineered Cell Line and Computational Docking Studies Reveals Enterotoxigenic *Escherichia coli* CFA/I Fimbriae Bind to Lewis a Glycans. Sci. Rep. 8, 11250. 10.1038/s41598-018-29258-0 30050155 PMC6062558

[B188] Ngo S. T. Hong N. D. Quynh Anh L. H. Hiep D. M. Tung N. T. (2020a). Effective Estimation of the Inhibitor Affinity of HIV-1 Protease via a Modified LIE Approach. RSC Adv. 10, 7732–7739. 10.1039/c9ra09583g PMC9049864 35492181

[B189] Ngo S. T. Mai B. K. Derreumaux P. Vu V. V. (2019). Adequate Prediction for Inhibitor Affinity of Aβ40 Protofibril Using the Linear Interaction Energy Method. RSC Adv. 9, 12455–12461. 10.1039/c9ra01177c PMC9063661 35515829

[B190] Ngo S. T. Quynh Anh Pham N. Thi Le L. Pham D.-H. Vu V. V. (2020b). Computational Determination of Potential Inhibitors of SARS-CoV-2 Main Protease. J. Chem. Inf. Model. 60, 5771–5780. 10.1021/acs.jcim.0c00491 32530282

[B191] Nishikawa N. Han K. Wu X. Tofoleanu F. Brooks B. R. (2018). Comparison of the Umbrella Sampling and the Double Decoupling Method in Binding Free Energy Predictions for SAMPL6 Octa-Acid Host-Guest Challenges. J. Comput. Aided Mol. Des. 32, 1075–1086. 10.1007/s10822-018-0166-2 30324304 PMC6413509

[B192] Ogunwa T. H. Laudadio E. Galeazzi R. Miyanishi T. (2019). Insights into the Molecular Mechanisms of Eg5 Inhibition by (+)-Morelloflavone. Pharmaceuticals (Basel) 12. 10.3390/ph12020058 PMC6630617 30995725

[B193] Öhlknecht C. Lier B. Petrov D. Fuchs J. Oostenbrink C. (2020a). Correcting Electrostatic Artifacts Due to Net‐charge Changes in the Calculation of Ligand Binding Free Energies. J. Comput. Chem. 41, 986–999. 10.1002/jcc.26143 31930547

[B194] Öhlknecht C. Perthold J. W. Lier B. Oostenbrink C. (2020b). Charge-Changing Perturbations and Path Sampling via Classical Molecular Dynamic Simulations of Simple Guest-Host Systems. J. Chem. Theor. Comput. 16, 7721–7734. 10.1021/acs.jctc.0c00719 PMC7726903 33136389

[B195] Okimoto N. Otsuka T. Hirano Y. Taiji M. (2018). Use of the Multilayer Fragment Molecular Orbital Method to Predict the Rank Order of Protein-Ligand Binding Affinities: A Case Study Using Tankyrase 2 Inhibitors. ACS Omega 3, 4475–4485. 10.1021/acsomega.8b00175 31458673 PMC6641631

[B196] Okiyama Y. Nakano T. Watanabe C. Fukuzawa K. Mochizuki Y. Tanaka S. (2018). Fragment Molecular Orbital Calculations with Implicit Solvent Based on the Poisson-Boltzmann Equation: Implementation and DNA Study. J. Phys. Chem. B 122, 4457–4471. 10.1021/acs.jpcb.8b01172 29558137

[B197] Okiyama Y. Watanabe C. Fukuzawa K. Mochizuki Y. Nakano T. Tanaka S. (2019). Fragment Molecular Orbital Calculations with Implicit Solvent Based on the Poisson-Boltzmann Equation: II. Protein and its Ligand-Binding System Studies. J. Phys. Chem. B 123, 957–973. 10.1021/acs.jpcb.8b09326 30532968

[B198] Ono F. Chiba S. Isaka Y. Matsumoto S. Ma B. Katayama R. (2020). Improvement in Predicting Drug Sensitivity Changes Associated with Protein Mutations Using a Molecular Dynamics Based Alchemical Mutation Method. Sci. Rep. 10, 2161. 10.1038/s41598-020-58877-9 32034220 PMC7005789

[B199] Ooi T. Oobatake M. Nemethy G. Scheraga H. A. (1987). Accessible Surface Areas as a Measure of the Thermodynamic Parameters of Hydration of Peptides. Proc. Natl. Acad. Sci. 84, 3086–3090. 10.1073/pnas.84.10.3086 3472198 PMC304812

[B200] Oshima H. Re S. Sugita Y. (2020). Prediction of Protein-Ligand Binding Pose and Affinity Using the gREST+FEP Method. J. Chem. Inf. Model. 60, 5382–5394. 10.1021/acs.jcim.0c00338 32786707

[B201] Pal R. K. Gallicchio E. (2019). Perturbation Potentials to Overcome Order/disorder Transitions in Alchemical Binding Free Energy Calculations. J. Chem. Phys. 151, 124116. 10.1063/1.5123154 31575187

[B202] Paliwal H. Shirts M. R. (2011). A Benchmark Test Set for Alchemical Free Energy Transformations and its Use to Quantify Error in Common Free Energy Methods. J. Chem. Theor. Comput. 7, 4115–4134. 10.1021/ct2003995 26598357

[B203] Pandey P. Srivastava R. Bandyopadhyay P. (2018). Comparison of Molecular Mechanics-Poisson-Boltzmann Surface Area (MM-PBSA) and Molecular Mechanics-Three-Dimensional Reference Interaction Site Model (MM-3d-RISM) Method to Calculate the Binding Free Energy of Protein-Ligand Complexes: Effect of Metal Ion and advance Statistical Test. Chem. Phys. Lett. 695, 69–78. 10.1016/j.cplett.2018.01.059

[B204] Patil R. Chikhale R. Khanal P. Gurav N. Ayyanar M. Sinha S. (2021). Computational and Network Pharmacology Analysis of Bioflavonoids as Possible Natural Antiviral Compounds in COVID-19. Inform. Med. Unlocked 22, 100504. 10.1016/j.imu.2020.100504 33363251 PMC7756171

[B205] Peng Y. Sun L. Jia Z. Li L. Alexov E. (2018). Predicting Protein-DNA Binding Free Energy Change upon Missense Mutations Using Modified MM/PBSA Approach: SAMPDI Webserver. Bioinformatics 34, 779–786. 10.1093/bioinformatics/btx698 29091991 PMC6048991

[B206] Pérez-Sánchez H. Thirumal Kumar D. George Priya Doss C. Rodríguez-Schmidt R. Cerón-Carrasco J. P. Peña-García J. (2021). Prediction and Characterization of Influenza Virus Polymerase Inhibitors through Blind Docking and Ligand Based Virtual Screening. J. Mol. Liquids 321. 10.1016/j.molliq.2020.114784

[B207] Perutz M. (1978). Electrostatic Effects in Proteins. Science 201, 1187–1191. 10.1126/science.694508 694508

[B208] Pham T. Nguyen H. L. Phan-Toai T. Nguyen H. (2020). Investigation of Binding Affinity between Potential Antiviral Agents and PB2 Protein of Influenza A: Non-equilibrium Molecular Dynamics Simulation Approach. Int. J. Med. Sci. 17, 2031–2039. 10.7150/ijms.46231 32788882 PMC7415388

[B209] Pham T. T. Shirts M. R. (2011). Identifying Low Variance Pathways for Free Energy Calculations of Molecular Transformations in Solution Phase. J. Chem. Phys. 135, 034114. 10.1063/1.3607597 21786994

[B210] Pourjafar-Dehkordi D. Vieweg S. Itzen A. Zacharias M. (2019). Phosphorylation of Ser111 in Rab8a Modulates Rabin8-dependent Activation by Perturbation of Side Chain Interaction Networks. Biochemistry 58, 3546–3554. 10.1021/acs.biochem.9b00516 31361120

[B211] Prabhakant A. Panigrahi A. Krishnan M. (2020). Allosteric Response of DNA Recognition Helices of Catabolite Activator Protein to cAMP and DNA Binding. J. Chem. Inf. Model. 60, 6366–6376. 10.1021/acs.jcim.0c00617 33108170

[B212] Procacci P. Macchiagodena M. Pagliai M. Guarnieri G. Iannone F. (2020). Interaction of Hydroxychloroquine with SARS-CoV2 Functional Proteins Using All-Atoms Non-equilibrium Alchemical Simulations. Chem. Commun. 56, 8854–8856. 10.1039/d0cc03558k 32633733

[B213] Proença C. Oliveira A. Freitas M. Ribeiro D. Sousa J. L. C. Ramos M. J. (2020). Structural Specificity of Flavonoids in the Inhibition of Human Fructose 1,6-Bisphosphatase. J. Nat. Prod. 83, 1541–1552. 10.1021/acs.jnatprod.0c00014 32364726

[B214] Qi R. Jing Z. Liu C. Piquemal J.-P. Dalby K. N. Ren P. (2018). Elucidating the Phosphate Binding Mode of Phosphate-Binding Protein: The Critical Effect of Buffer Solution. J. Phys. Chem. B 122, 6371–6376. 10.1021/acs.jpcb.8b03194 29807433 PMC6173968

[B215] Qi R. Luo R. (2019). Robustness and Efficiency of Poisson-Boltzmann Modeling on Graphics Processing Units. J. Chem. Inf. Model. 59, 409–420. 10.1021/acs.jcim.8b00761 30550277 PMC6430105

[B216] Qi R. Walker B. Jing Z. Yu M. Stancu G. Edupuganti R. (2019). Computational and Experimental Studies of Inhibitor Design for Aldolase A. J. Phys. Chem. B 123, 6034–6041. 10.1021/acs.jpcb.9b04551 31268712 PMC6935369

[B217] Qian Y. Cabeza De Vaca I. Vilseck J. Z. Cole D. J. Tirado-Rives J. Jorgensen W. L. (2019). Absolute Free Energy of Binding Calculations for Macrophage Migration Inhibitory Factor in Complex with a Druglike Inhibitor. J. Phys. Chem. B 123, 8675–8685. 10.1021/acs.jpcb.9b07588 31553604 PMC7932129

[B218] Rabbad A. H. Agoni C. Olotu F. A. Soliman M. E. (2019). Microbes, Not Humans: Exploring the Molecular Basis of Pseudouridimycin Selectivity towards Bacterial and Not Human RNA Polymerase. Biotechnol. Lett. 41, 115–128. 10.1007/s10529-018-2617-1 30377869

[B219] Rahman S. U. Ali H. S. Jafari B. Zaib S. Hameed A. Al-Kahraman Y. (2020). Structure-based Virtual Screening of Dipeptidyl Peptidase 4 Inhibitors and Their *In Vitro* Analysis. Comput. Biol. Chem., 107326. 32739275 10.1016/j.compbiolchem.2020.107326

[B220] Rana R. M. Rampogu S. Abid N. B. Zeb A. Parate S. Lee G. (2020). Silico Study Identified Methotrexate Analog as Potential Inhibitor of Drug Resistant Human Dihydrofolate Reductase for Cancer Therapeutics. Molecules 25. 10.3390/molecules25153510 PMC7435474 32752079

[B221] Rastelli G. Del Rio A. Degliesposti G. Sgobba M. (2010). Fast and Accurate Predictions of Binding Free Energies Using MM-PBSA and MM-GBSA. J. Comput. Chem. 31, 797–810. 10.1002/jcc.21372 19569205

[B222] Reis P. B. P. S. Vila-Viçosa D. Rocchia W. Machuqueiro M. (2020). PypKa: A Flexible Python Module for Poisson-Boltzmann-Based pKa Calculations. J. Chem. Inf. Model. 60, 4442–4448. 10.1021/acs.jcim.0c00718 32857502

[B223] Ricci-Lopez J. Vidal-Limon A. Zunniga M. Jimenez V. A. Alderete J. B. Brizuela C. A. (2019). Molecular Modeling Simulation Studies Reveal New Potential Inhibitors against HPV E6 Protein. PLoS One 14, e0213028. 10.1371/journal.pone.0213028 30875378 PMC6420176

[B224] Rifai E. A. Ferrario V. Pleiss J. Geerke D. P. (2020). Combined Linear Interaction Energy and Alchemical Solvation Free-Energy Approach for Protein-Binding Affinity Computation. J. Chem. Theor. Comput. 16, 1300–1310. 10.1021/acs.jctc.9b00890 PMC7017367 31894691

[B225] Rifai E. A. Van Dijk M. Vermeulen N. P. E. Yanuar A. Geerke D. P. (2019). A Comparative Linear Interaction Energy and MM/PBSA Study on SIRT1-Ligand Binding Free Energy Calculation. J. Chem. Inf. Model. 59, 4018–4033. 10.1021/acs.jcim.9b00609 31461271 PMC6759767

[B226] Rizzi A. Jensen T. Slochower D. R. Aldeghi M. Gapsys V. Ntekoumes D. (2020). The SAMPL6 SAMPLing challenge: Assessing the Reliability and Efficiency of Binding Free Energy Calculations. J. Comput. Aided Mol. Des. 34, 601–633. 10.1007/s10822-020-00290-5 31984465 PMC7282318

[B227] Rocklin G. J. Mobley D. L. Dill K. A. Hünenberger P. H. (2013). Calculating the Binding Free Energies of Charged Species Based on Explicit-Solvent Simulations Employing Lattice-Sum Methods: an Accurate Correction Scheme for Electrostatic Finite-Size Effects. J. Chem. Phys. 139, 184103. 10.1063/1.4826261 24320250 PMC3838431

[B228] Roux B. Nina M. Pomès R. Smith J. C. (1996). Thermodynamic Stability of Water Molecules in the Bacteriorhodopsin Proton Channel: a Molecular Dynamics Free Energy Perturbation Study. Biophysical J. 71, 670–681. 10.1016/s0006-3495(96)79267-6 PMC1233524 8842206

[B229] Ryberg L. A. Sønderby P. Bukrinski J. T. Harris P. Peters G. H. J. (2020). Investigations of Albumin-Insulin Detemir Complexes Using Molecular Dynamics Simulations and Free Energy Calculations. Mol. Pharmaceutics 17, 132–144. 10.1021/acs.molpharmaceut.9b00839 31790268

[B230] Sabri M. Z. Abdul Hamid A. A. Sayed Hitam S. M. Abdul Rahim M. Z. (2019). Silico Screening of Aptamers Configuration against Hepatitis B Surface Antigen. Adv. Bioinformatics 2019, 6912914. 10.1155/2019/6912914 31346332 PMC6617924

[B231] Sahoo A. R. Mishra R. Rana S. (2018). The Model Structures of the Complement Component 5a Receptor (C5aR) Bound to the Native and Engineered (h)C5a. Sci. Rep. 8, 2955. 10.1038/s41598-018-21290-4 29440703 PMC5811428

[B232] Sakae Y. Zhang B. W. Levy R. M. Deng N. (2020). Absolute Protein Binding Free Energy Simulations for Ligands with Multiple Poses, a Thermodynamic Path that Avoids Exhaustive Enumeration of the Poses. J. Comput. Chem. 41, 56–68. 10.1002/jcc.26078 31621932 PMC7140983

[B233] Sakkiah S. Guo W. Pan B. Ji Z. Yavas G. Azevedo M. (2020). Elucidating Interactions between SARS-CoV-2 Trimeric Spike Protein and ACE2 Using Homology Modeling and Molecular Dynamics Simulations. Front. Chem. 8, 622632. 10.3389/fchem.2020.622632 33469527 PMC7813797

[B234] Schaller K. S. Kari J. Molina G. A. Tidemand K. D. Borch K. Peters G. H. J. (2021). Computing Cellulase Kinetics with a Two-Domain Linear Interaction Energy Approach. ACS Omega 6, 1547–1555. 10.1021/acsomega.0c05361 33490814 PMC7818601

[B235] Senapathi T. Suruzhon M. Barnett C. B. Essex J. Naidoo K. J. (2020). BRIDGE: An Open Platform for Reproducible High-Throughput Free Energy Simulations. J. Chem. Inf. Model. 60, 5290–5295. 10.1021/acs.jcim.0c00206 32810405

[B236] Serapian S. A. Van Der Kamp M. W. (2019). Unpicking the Cause of Stereoselectivity in Actinorhodin Ketoreductase Variants with Atomistic Simulations. ACS Catal. 9, 2381–2394. 10.1021/acscatal.8b04846

[B237] Shahbaaz M. Nkaule A. Christoffels A. (2019). Designing Novel Possible Kinase Inhibitor Derivatives as Therapeutics against *Mycobacterium tuberculosis*: An In Silico Study. Sci. Rep. 9, 4405. 10.1038/s41598-019-40621-7 30867456 PMC6416319

[B238] Shaker B. Ahmad S. Thai T. D. Eyun S. I. Na D. (2020). Rational Drug Design for *Pseudomonas aeruginosa* PqsA Enzyme: An In Silico Guided Study to Block Biofilm Formation. Front. Mol. Biosci. 7, 577316. 10.3389/fmolb.2020.577316 33195420 PMC7593710

[B239] Shao Q. Zhu W. (2019). Exploring the Ligand Binding/Unbinding Pathway by Selectively Enhanced Sampling of Ligand in a Protein-Ligand Complex. J. Phys. Chem. B 123, 7974–7983. 10.1021/acs.jpcb.9b05226 31478672

[B240] Sharp K. A. Honig B. (2002). Calculating Total Electrostatic Energies with the Nonlinear Poisson-Boltzmann Equation. J. Phys. Chem. 94, 7684–7692. 10.1021/j100382a068

[B241] Sharp K. A. Honig B. (1990). Electrostatic Interactions in Macromolecules: Theory and Applications. Annu. Rev. Biophys. Biophys. Chem. 19, 301–332. 10.1146/annurev.bb.19.060190.001505 2194479

[B242] Shen L. Yuan Y. Guo Y. Li M. Li C. Pu X. (2019). Probing the Druggablility on the Interface of the Protein-Protein Interaction and its Allosteric Regulation Mechanism on the Drug Screening for the CXCR4 Homodimer. Front. Pharmacol. 10, 1310. 10.3389/fphar.2019.01310 31787895 PMC6855241

[B243] Shi Y. Laury M. L. Wang Z. Ponder J. W. (2020). AMOEBA Binding Free Energies for the SAMPL7 TrimerTrip Host-Guest challenge. J. Comput. Aided Mol. Des. 35, 79–93. 10.1007/s10822-020-00358-2 33140208 PMC7867568

[B244] Shirts M. R. (2012). Best Practices in Free Energy Calculations for Drug Design. Methods Mol. Biol. 819, 425–467. 10.1007/978-1-61779-465-0_26 22183551

[B245] Shirts M. R. Chodera J. D. (2008). Statistically Optimal Analysis of Samples from Multiple Equilibrium States. J. Chem. Phys. 129, 124105. 10.1063/1.2978177 19045004 PMC2671659

[B246] Shirts M. R. Pande V. S. (2005). Comparison of Efficiency and Bias of Free Energies Computed by Exponential Averaging, the Bennett Acceptance Ratio, and Thermodynamic Integration. J. Chem. Phys. 122, 144107. 10.1063/1.1873592 15847516

[B247] Shuaib S. Narang S. S. Goyal D. Goyal B. (2019). Computational Design and Evaluation of β‐sheet Breaker Peptides for Destabilizing Alzheimer's Amyloid‐β 42 Protofibrils. J. Cel Biochem 120, 17935–17950. 10.1002/jcb.29061 31162715

[B248] Singh N. Li W. (2020). Absolute Binding Free Energy Calculations for Highly Flexible Protein MDM2 and its Inhibitors. Int. J. Mol. Sci. 21. 10.3390/ijms21134765 PMC7369993 32635537

[B249] Singh R. Gautam A. Chandel S. Ghosh A. Dey D. Roy S. (2020). Protease Inhibitory Effect of Natural Polyphenolic Compounds on SARS-CoV-2: An In Silico Study. Molecules 25. 10.3390/molecules25204604 PMC7587198 33050360

[B250] Sk M. F. Jonniya N. A. Roy R. Poddar S. Kar P. (2020). Computational Investigation of Structural Dynamics of SARS-CoV-2 Methyltransferase-Stimulatory Factor Heterodimer Nsp16/nsp10 Bound to the Cofactor SAM. Front. Mol. Biosci. 7, 590165. 10.3389/fmolb.2020.590165 33330626 PMC7732651

[B251] Slynko I. Scharfe M. Rumpf T. Eib J. Metzger E. Schüle R. (2014). Virtual Screening of PRK1 Inhibitors: Ensemble Docking, Rescoring Using Binding Free Energy Calculation and QSAR Model Development. J. Chem. Inf. Model. 54, 138–150. 10.1021/ci400628q 24377786

[B252] Song L. F. Merz K. M. Jr. (2020). Evolution of Alchemical Free Energy Methods in Drug Discovery. J. Chem. Inf. Model. 60, 5308–5318. 10.1021/acs.jcim.0c00547 32818371

[B253] Song L. F. Sengupta A. Merz K. M. Jr. (2020). Thermodynamics of Transition Metal Ion Binding to Proteins. J. Am. Chem. Soc. 142, 6365–6374. 10.1021/jacs.0c01329 32141296

[B254] Srinivasan J. Cheatham T. E. Cieplak P. Kollman P. A. Case D. A. (1998). Continuum Solvent Studies of the Stability of DNA, RNA, and Phosphoramidate−DNA Helices. J. Am. Chem. Soc. 120, 9401–9409. 10.1021/ja981844+

[B255] Steinbrecher T. Joung I. Case D. A. (2011). Soft-core Potentials in Thermodynamic Integration: Comparing One- and Two-step Transformations. J. Comput. Chem. 32, 3253–3263. 10.1002/jcc.21909 21953558 PMC3187911

[B256] Steinbrecher T. Mobley D. L. Case D. A. (2007). Nonlinear Scaling Schemes for Lennard-Jones Interactions in Free Energy Calculations. J. Chem. Phys. 127, 214108. 10.1063/1.2799191 18067350

[B257] Straatsma T. P. Mccammon J. A. (1991). Multiconfiguration Thermodynamic Integration. J. Chem. Phys. 95, 1175–1188. 10.1063/1.461148

[B258] Su L. Wang Y. Wang J. Mifune Y. Morin M. D. Jones B. T. (2019). Structural Basis of TLR2/TLR1 Activation by the Synthetic Agonist Diprovocim. J. Med. Chem. 62, 2938–2949. 10.1021/acs.jmedchem.8b01583 30829478 PMC6537610

[B259] Sun H. Duan L. Chen F. Liu H. Wang Z. Pan P. (2018). Assessing the Performance of MM/PBSA and MM/GBSA Methods. 7. Entropy Effects on the Performance of End-point Binding Free Energy Calculation Approaches. Phys. Chem. Chem. Phys. 20, 14450–14460. 10.1039/c7cp07623a 29785435

[B260] Sun H. Li Y. Shen M. Tian S. Xu L. Pan P. (2014). Assessing the Performance of MM/PBSA and MM/GBSA Methods. 5. Improved Docking Performance Using High Solute Dielectric Constant MM/GBSA and MM/PBSA Rescoring. Phys. Chem. Chem. Phys. 16, 22035–22045. 10.1039/c4cp03179b 25205360

[B261] Swanson J. M. J. Henchman R. H. Mccammon J. A. (2004). Revisiting Free Energy Calculations: a Theoretical Connection to MM/PBSA and Direct Calculation of the Association Free Energy. Biophysical J. 86, 67–74. 10.1016/s0006-3495(04)74084-9 PMC1303837 14695250

[B262] Tafazzol A. Duan Y. (2019). Key Residues in TLR4-MD2 Tetramer Formation Identified by Free Energy Simulations. Plos Comput. Biol. 15, e1007228. 10.1371/journal.pcbi.1007228 31609969 PMC6812856

[B263] Tan C. Tan Y.-H. Luo R. (2007). Implicit Nonpolar Solvent Models. J. Phys. Chem. B 111, 12263–12274. 10.1021/jp073399n 17918880

[B264] Tan C. Yang L. Luo R. (2006). How Well Does Poisson−Boltzmann Implicit Solvent Agree with Explicit Solvent? A Quantitative Analysis. J. Phys. Chem. B 110, 18680–18687. 10.1021/jp063479b 16970499

[B265] Tanida Y. Matsuura A. (2020). Alchemical Free Energy Calculations via Metadynamics: Application to the theophylline‐RNA Aptamer Complex. J. Comput. Chem. 41, 1804–1819. 10.1002/jcc.26221 32449538

[B266] Tavakoli F. Ganjalikhany M. R. (2019). Structure-based Inhibitory Peptide Design Targeting Peptide-Substrate Binding Site in EGFR Tyrosine Kinase. PLoS One 14, e0217031. 10.1371/journal.pone.0217031 31116768 PMC6530890

[B267] Tejera E. Munteanu C. R. Lopez-Cortes A. Cabrera-Andrade A. Perez-Castillo Y. (2020). Drugs Repurposing Using QSAR, Docking and Molecular Dynamics for Possible Inhibitors of the SARS-CoV-2 M(pro) Protease. Molecules 25. 10.3390/molecules25215172 PMC7664330 33172092

[B268] Terayama K. Iwata H. Araki M. Okuno Y. Tsuda K. (2018). Machine Learning Accelerates MD-based Binding Pose Prediction between Ligands and Proteins. Bioinformatics 34, 770–778. 10.1093/bioinformatics/btx638 29040432 PMC6030886

[B269] Thai N. Q. Bednarikova Z. Gancar M. Linh H. Q. Hu C.-K. Li M. S. (2018). Compound CID 9998128 Is a Potential Multitarget Drug for Alzheimer's Disease. ACS Chem. Neurosci. 9, 2588–2598. 10.1021/acschemneuro.8b00091 29775277

[B270] Tian X. Liu Y. Zhu J. Yu Z. Han J. Wang Y. (2018). Probing Inhibition Mechanisms of Adenosine Deaminase by Using Molecular Dynamics Simulations. PLoS One 13, e0207234. 10.1371/journal.pone.0207234 30444912 PMC6239307

[B271] Tzitzoglaki C. Mcguire K. Lagarias P. Konstantinidi A. Hoffmann A. Fokina N. A. (2020). Chemical Probes for Blocking of Influenza A M2 Wild-type and S31N Channels. ACS Chem. Biol. 15, 2331–2337. 10.1021/acschembio.0c00553 32786258 PMC8051587

[B272] Van Dijk M. Ter Laak A. M. Wichard J. D. Capoferri L. Vermeulen N. P. E. Geerke D. P. (2017). Comprehensive and Automated Linear Interaction Energy Based Binding-Affinity Prediction for Multifarious Cytochrome P450 Aromatase Inhibitors. J. Chem. Inf. Model. 57, 2294–2308. 10.1021/acs.jcim.7b00222 28776988 PMC5615371

[B273] Vargas R. E. Duong V. T. Han H. Ta A. P. Chen Y. Zhao S. (2020). Elucidation of WW Domain Ligand Binding Specificities in the Hippo Pathway Reveals STXBP4 as YAP Inhibitor. EMBO J. 39, e102406. 10.15252/embj.2019102406 31782549 PMC6939200

[B274] Venugopala K. N. Chandrashekharappa S. Pillay M. Abdallah H. H. Mahomoodally F. M. Bhandary S. (2019). Computational, Crystallographic Studies, Cytotoxicity and Anti-tubercular Activity of Substituted 7-Methoxy-Indolizine Analogues. PLoS One 14, e0217270. 10.1371/journal.pone.0217270 31163040 PMC6548424

[B275] Vongsutilers V. Shinohara Y. Kawai G. (2020). Epigenetic TET-Catalyzed Oxidative Products of 5-Methylcytosine Impede Z-DNA Formation of CG Decamers. ACS Omega 5, 8056–8064. 10.1021/acsomega.0c00120 32309715 PMC7161056

[B276] Wagoner J. A. Baker N. A. (2006). Assessing Implicit Models for Nonpolar Mean Solvation Forces: the Importance of Dispersion and Volume Terms. Proc. Natl. Acad. Sci. 103, 8331–8336. 10.1073/pnas.0600118103 16709675 PMC1482494

[B277] Wakchaure P. D. Ghosh S. Ganguly B. (2020). Revealing the Inhibition Mechanism of RNA-dependent RNA Polymerase (RdRp) of SARS-CoV-2 by Remdesivir and Nucleotide Analogues: A Molecular Dynamics Simulation Study. J. Phys. Chem. B 124, 10641–10652. 10.1021/acs.jpcb.0c06747 33190493

[B278] Walker B. Jing Z. Ren P. (2020). Molecular Dynamics Free Energy Simulations of ATP:Mg2+ and ADP:Mg2+ Using the Polarisable Force Field AMOEBA. Mol. Simulation, 1–10. 10.1080/08927022.2020.1725003 PMC8375395 34421214

[B279] Wallace J. A. Shen J. K. (2012). Charge-leveling and Proper Treatment of Long-Range Electrostatics in All-Atom Molecular Dynamics at Constant pH. J. Chem. Phys. 137, 184105. 10.1063/1.4766352 23163362 PMC3511335

[B280] Wang C. Greene D. Xiao L. Qi R. Luo R. (2018a). Recent Developments and Applications of the MMPBSA Method. Front. Mol. Biosci. 4, 87. 10.3389/fmolb.2017.00087 29367919 PMC5768160

[B281] Wang C. Nguyen P. H. Pham K. Huynh D. Le T.-B. N. Wang H. (2016). Calculating Protein-Ligand Binding Affinities with MMPBSA: Method and Error Analysis. J. Comput. Chem. 37, 2436–2446. 10.1002/jcc.24467 27510546 PMC5018451

[B282] Wang C. Wang J. Cai Q. Li Z. Zhao H.-K. Luo R. (2013). Exploring Accurate Poisson-Boltzmann Methods for Biomolecular Simulations. Comput. Theor. Chem. 1024, 34–44. 10.1016/j.comptc.2013.09.021 24443709 PMC3891588

[B283] Wang C. Xiao L. Luo R. (2017). Numerical Interpretation of Molecular Surface Field in Dielectric Modeling of Solvation. J. Comput. Chem. 38, 1057–1070. 10.1002/jcc.24782 28318096 PMC5464005

[B284] Wang E. Sun H. Wang J. Wang Z. Liu H. Zhang J. Z. H. (2019a). End-Point Binding Free Energy Calculation with MM/PBSA and MM/GBSA: Strategies and Applications in Drug Design. Chem. Rev. 119, 9478–9508. 10.1021/acs.chemrev.9b00055 31244000

[B285] Wang E. Weng G. Sun H. Du H. Zhu F. Chen F. (2019b). Assessing the Performance of the MM/PBSA and MM/GBSA Methods. 10. Impacts of Enhanced Sampling and Variable Dielectric Model on Protein-Protein Interactions. Phys. Chem. Chem. Phys. 21, 18958–18969. 10.1039/c9cp04096j 31453590

[B286] Wang J. Luo R. (2010). Assessment of Linear Finite-Difference Poisson-Boltzmann Solvers. J. Comput. Chem. 31, 1689–1698. 10.1002/jcc.21456 20063271 PMC2854862

[B287] Wang J. Cai Q. Li Z.-L. Zhao H.-K. Luo R. (2009). Achieving Energy Conservation in Poisson-Boltzmann Molecular Dynamics: Accuracy and Precision with Finite-Difference Algorithms. Chem. Phys. Lett. 468, 112–118. 10.1016/j.cplett.2008.12.049 20098487 PMC2663913

[B288] Wang J. Cai Q. Xiang Y. Luo R. (2012). Reducing Grid Dependence in Finite-Difference Poisson-Boltzmann Calculations. J. Chem. Theor. Comput. 8, 2741–2751. 10.1021/ct300341d PMC3505068 23185142

[B289] Wang J. Tan C. Chanco E. Luo R. (2010). Quantitative Analysis of Poisson-Boltzmann Implicit Solvent in Molecular Dynamics. Phys. Chem. Chem. Phys. 12, 1194–1202. 10.1039/b917775b 20094685

[B290] Wang K. Huang Q. Li H. Zhao X. (2020a). Co-evolution of β-glucosidase Activity and Product Tolerance for Increasing Cellulosic Ethanol Yield. Biotechnol. Lett. 42, 2239–2250. 10.1007/s10529-020-02935-9 32583369

[B291] Wang L. Wu Y. Deng Y. Kim B. Pierce L. Krilov G. (2015). Accurate and Reliable Prediction of Relative Ligand Binding Potency in Prospective Drug Discovery by Way of a Modern Free-Energy Calculation Protocol and Force Field. J. Am. Chem. Soc. 137, 2695–2703. 10.1021/ja512751q 25625324

[B292] Wang R.-G. Zhang H.-X. Zheng Q.-C. (2020b). Revealing the Binding and Drug Resistance Mechanism of Amprenavir, Indinavir, Ritonavir, and Nelfinavir Complexed with HIV-1 Protease Due to Double Mutations G48T/L89M by Molecular Dynamics Simulations and Free Energy Analyses. Phys. Chem. Chem. Phys. 22, 4464–4480. 10.1039/c9cp06657h 32057044

[B293] Wang R. Tao W. Liu L. Li C. Bai L. Zhao Y. L. (2021). Insights into Specificity and Catalytic Mechanism of Amphotericin B/nystatin Thioesterase. Proteins. 89, 558–568. 10.1002/prot.26041 33389775

[B294] Wang R. Zheng Q. (2020). Multiple Molecular Dynamics Simulations of the Inhibitor GRL-02031 Complex with Wild Type and Mutant HIV-1 Protease Reveal the Binding and Drug-Resistance Mechanism. Langmuir 36, 13817–13832. 10.1021/acs.langmuir.0c02151 33175558

[B295] Wang W. Wang J. Kollman P. A. (1999). What determines the van der Waals coefficient ? in the LIE (linear interaction energy) method to estimate binding free energies using molecular dynamics simulations? Proteins 34, 395–402. 10.1002/(sici)1097-0134(19990215)34:3<395::aid-prot11>3.0.co;2-4 10024025

[B296] Wang Y. Liu J. Li J. He X. (2018b). Fragment-based Quantum Mechanical Calculation of Protein-Protein Binding Affinities. J. Comput. Chem. 39, 1617–1628. 10.1002/jcc.25236 29707784

[B297] Wang Z. Wang X. Li Y. Lei T. Wang E. Li D. (2019c). farPPI: a Webserver for Accurate Prediction of Protein-Ligand Binding Structures for Small-Molecule PPI Inhibitors by MM/PB(GB)SA Methods. Bioinformatics 35, 1777–1779. 10.1093/bioinformatics/bty879 30329012

[B298] Warwicker J. Watson H. C. (1982). Calculation of the Electric Potential in the Active Site Cleft Due to α-helix Dipoles. J. Mol. Biol. 157, 671–679. 10.1016/0022-2836(82)90505-8 6288964

[B299] Wäschenbach L. Gertzen C. G. W. Keitel V. Gohlke H. (2020). Dimerization Energetics of the G‐protein Coupled Bile Acid Receptor TGR5 from All‐atom Simulations. J. Comput. Chem. 41, 874–884. 10.1002/jcc.26135 31880348

[B300] Wei H. Luo A. Qiu T. Luo R. Qi R. (2019a). Improved Poisson-Boltzmann Methods for High-Performance Computing. J. Chem. Theor. Comput. 15, 6190–6202. 10.1021/acs.jctc.9b00602 PMC7194241 31525962

[B301] Wei H. Luo R. Qi R. (2019b). An Efficient Second‐order Poisson-Boltzmann Method. J. Comput. Chem. 40, 1257–1269. 10.1002/jcc.25783 30776135 PMC6422926

[B302] Wei Y. Zhang M. Jiao P. Zhang X. Yang G. Xu X. (2021). Intracellular Paclitaxel Delivery Facilitated by a Dual-Functional CPP with a Hydrophobic Hairpin Tail. ACS Appl. Mater. Inter. 13, 4853–4860. 10.1021/acsami.0c20180 33474938

[B303] Womack J. C. Anton L. Dziedzic J. Hasnip P. J. Probert M. I. J. Skylaris C.-K. (2018). DL_MG: A Parallel Multigrid Poisson and Poisson-Boltzmann Solver for Electronic Structure Calculations in Vacuum and Solution. J. Chem. Theor. Comput. 14, 1412–1432. 10.1021/acs.jctc.7b01274 29447447

[B304] Woo H.-J. Roux B. (2005). Calculation of Absolute Protein-Ligand Binding Free Energy from Computer Simulations. Proc. Natl. Acad. Sci. 102, 6825–6830. 10.1073/pnas.0409005102 15867154 PMC1100764

[B305] Wright D. W. Wan S. Meyer C. Van Vlijmen H. Tresadern G. Coveney P. V. (2019). Application of ESMACS Binding Free Energy Protocols to Diverse Datasets: Bromodomain-Containing Protein 4. Sci. Rep. 9, 6017. 10.1038/s41598-019-41758-1 30979914 PMC6461631

[B306] Xia J. Flynn W. Gallicchio E. Uplinger K. Armstrong J. D. Forli S. (2019). Massive-Scale Binding Free Energy Simulations of HIV Integrase Complexes Using Asynchronous Replica Exchange Framework Implemented on the IBM WCG Distributed Network. J. Chem. Inf. Model. 59, 1382–1397. 10.1021/acs.jcim.8b00817 30758197 PMC6496938

[B307] Xiao Z. Cong Y. Huang K. Zhong S. Zhang J. Z. H. Duan L. (2019). Drug-resistance Mechanisms of Three Mutations in Anaplastic Lymphoma Kinase against Two Inhibitors Based on MM/PBSA Combined with Interaction Entropy. Phys. Chem. Chem. Phys. 21, 20951–20964. 10.1039/c9cp02851j 31524891

[B308] Xue J. Huang X. Zhu Y. (2019). Using Molecular Dynamics Simulations to Evaluate Active Designs of Cephradine Hydrolase by Molecular mechanics/Poisson-Boltzmann Surface Area and Molecular Mechanics/generalized Born Surface Area Methods. RSC Adv. 9, 13868–13877. 10.1039/c9ra02406a PMC9064048 35519543

[B309] Yang J.-F. Williams A. H. Penthala N. R. Prather P. L. Crooks P. A. Zhan C.-G. (2020). Binding Modes and Selectivity of Cannabinoid 1 (CB1) and Cannabinoid 2 (CB2) Receptor Ligands. ACS Chem. Neurosci. 11, 3455–3463. 10.1021/acschemneuro.0c00551 32997485 PMC7756905

[B310] Yang T. Wu J. C. Yan C. Wang Y. Luo R. Gonzales M. B. (2011). Virtual Screening Using Molecular Simulations. Proteins 79, 1940–1951. 10.1002/prot.23018 21491494 PMC3092865

[B311] Yang W. Bitetti-Putzer R. Karplus M. (2004). Free Energy Simulations: Use of Reverse Cumulative Averaging to Determine the Equilibrated Region and the Time Required for Convergence. J. Chem. Phys. 120, 2618–2628. 10.1063/1.1638996 15268405

[B312] Yau M. Q. Emtage A. L. Chan N. J. Y. Doughty S. W. Loo J. S. E. (2019). Evaluating the Performance of MM/PBSA for Binding Affinity Prediction Using Class A GPCR crystal Structures. J. Comput. Aided Mol. Des. 33, 487–496. 10.1007/s10822-019-00201-3 30989574

[B313] Yau M. Q. Emtage A. L. Loo J. S. E. (2020). Benchmarking the Performance of MM/PBSA in Virtual Screening Enrichment Using the GPCR-Bench Dataset. J. Comput. Aided Mol. Des. 34, 1133–1145. 10.1007/s10822-020-00339-5 32851579

[B314] Ye X. Cai Q. Yang W. Luo R. (2009). Roles of Boundary Conditions in DNA Simulations: Analysis of Ion Distributions with the Finite-Difference Poisson-Boltzmann Method. Biophysical J. 97, 554–562. 10.1016/j.bpj.2009.05.012 PMC2711334 19619470

[B315] Ye X. Wang J. Luo R. (2010). A Revised Density Function for Molecular Surface Calculation in Continuum Solvent Models. J. Chem. Theor. Comput. 6, 1157–1169. 10.1021/ct900318u PMC3979486 24723844

[B316] Yu R. Cheng L. P. Li M. Pang W. (2019). Discovery of Novel Neuraminidase Inhibitors by Structure-Based Virtual Screening, Structural Optimization, and Bioassay. ACS Med. Chem. Lett. 10, 1667–1673. 10.1021/acsmedchemlett.9b00447 31857844 PMC6912870

[B317] Zaman Z. Khan S. Nouroz F. Farooq U. Urooj A. (2021). Targeting Protein Tyrosine Phosphatase to Unravel Possible Inhibitors for Streptococcus Pneumoniae Using Molecular Docking, Molecular Dynamics Simulations Coupled with Free Energy Calculations. Life Sci. 264, 118621. 10.1016/j.lfs.2020.118621 33164832

[B318] Zampieri D. Fortuna S. Calabretti A. Romano M. Menegazzi R. Schepmann D. (2020). Synthesis, Cytotoxicity Evaluation, and Computational Insights of Novel 1,4-Diazepane-Based Sigma Ligands. ACS Med. Chem. Lett. 11, 651–656. 10.1021/acsmedchemlett.9b00524 32435366 PMC7236030

[B319] Zasada S. J. Wright D. W. Coveney P. V. (2020). Large-scale Binding Affinity Calculations on Commodity Compute Clouds. Interf. Focus. 10, 20190133. 10.1098/rsfs.2019.0133 PMC7653340 33178415

[B320] Zazeri G. Povinelli A. P. R. Lima M. F. Cornelio M. L. (2020). Detailed Characterization of the Cooperative Binding of Piperine with Heat Shock Protein 70 by Molecular Biophysical Approaches. Biomedicines 8. 10.3390/biomedicines8120629 PMC7766160 33353024

[B321] Zhang B. D’Erasmo M. P. Murelli R. P. Gallicchio E. (2016). Free Energy-Based Virtual Screening and Optimization of RNase H Inhibitors of HIV-1 Reverse Transcriptase. ACS Omega 1, 435–447. 10.1021/acsomega.6b00123 27713931 PMC5046171

[B322] Zhang H. Gattuso H. Dumont E. Cai W. Monari A. Chipot C. (2018). Accurate Estimation of the Standard Binding Free Energy of Netropsin with DNA. Molecules 23. 10.3390/molecules23020228 PMC6017086 29370096

[B323] Zhang H. Jiang W. Chatterjee P. Luo Y. (2019a). Ranking Reversible Covalent Drugs: From Free Energy Perturbation to Fragment Docking. J. Chem. Inf. Model. 59, 2093–2102. 10.1021/acs.jcim.8b00959 30763080 PMC6610880

[B324] Zhang J.-L. Liu X. Zhang H.-X. (2020). Interaction Mechanism of the Germination Stimulants Karrikins and Their Receptor ShKAI2iB. J. Phys. Chem. B 124, 9812–9819. 10.1021/acs.jpcb.0c06734 33089685

[B325] Zhang X. Sun H. Wen X. Yuan H. (2019b). A Selectivity Study of FFAR4/FFAR1 Agonists by Molecular Modeling. J. Chem. Inf. Model. 59, 4467–4474. 10.1021/acs.jcim.9b00735 31580060

[B326] Zhao S. Ni F. Qiu T. Wolff J. T. Tsai S. C. Luo R. (2020). Molecular Basis for Polyketide Ketoreductase-Substrate Interactions. Int. J. Mol. Sci. 21. 10.3390/ijms21207562 PMC7588967 33066287

[B327] Zhou X. Zheng J. Ivan F. X. Yin R. Ranganathan S. Chow V. T. K. (2018). Computational Analysis of the Receptor Binding Specificity of Novel Influenza A/H7N9 Viruses. BMC Genomics 19, 88. 10.1186/s12864-018-4461-z 29764421 PMC5954268

[B328] Zwanzig R. W. (1954). High‐Temperature Equation of State by a Perturbation Method. I. Nonpolar Gases. J. Chem. Phys. 22, 1420–1426. 10.1063/1.1740409

